# Population Structure and Evolution of *Rhinoviruses*

**DOI:** 10.1371/journal.pone.0088981

**Published:** 2014-02-19

**Authors:** Vaishali P. Waman, Pandurang S. Kolekar, Mohan M. Kale, Urmila Kulkarni-Kale

**Affiliations:** 1 Bioinformatics Centre, University of Pune, Pune, India; 2 Department of Statistics, University of Pune, Pune, India; North Carolina State University, United States of America

## Abstract

*Rhinoviruses,* formerly known as *Human rhinoviruses*, are the most common cause of air-borne upper respiratory tract infections in humans. *Rhinoviruses* belong to the family *Picornaviridae* and are divided into three species namely, *Rhinovirus A, -B* and -*C*, which are antigenically diverse. Genetic recombination is found to be one of the important causes for diversification of *Rhinovirus* species. Although emerging lineages within *Rhinoviruses* have been reported, their population structure has not been studied yet. The availability of complete genome sequences facilitates study of population structure, genetic diversity and underlying evolutionary forces, such as mutation, recombination and selection pressure. Analysis of complete genomes of *Rhinoviruses* using a model-based population genetics approach provided a strong evidence for existence of seven genetically distinct subpopulations. As a result of diversification, *Rhinovirus A* and *-C* populations are divided into four and two subpopulations, respectively. Genetically, the *Rhinovirus B* population was found to be homogeneous. Intra-species recombination was observed to be prominent in *Rhinovirus A* and *-C* species. Significant evidence of episodic positive selection was obtained for several sites within coding sequences of structural and non-structural proteins. This corroborates well with known phenotypic properties such as antigenicity of structural proteins. Episodic positive selection appears to be responsible for emergence of new lineages especially in *Rhinovirus A*. In summary, the *Rhinovirus* population is an ensemble of seven distinct lineages. In case of *Rhinovirus A,* intra-species recombination and episodic positive selection contribute to its further diversification. In case of *Rhinovirus C,* intra- and inter-species recombinations are responsible for observed diversity. Population genetics approach was further useful to analyze phylogenetic tree topologies pertaining to recombinant strains, especially when trees are derived using complete genomes. Understanding of population structure serves as a foundation for designing new vaccines and drugs as well as to explain emergence of drug resistance amongst subpopulations.

## Introduction

*Rhinoviruses* are known to infect upper and lower respiratory tract and cause common cold in humans. Though common cold is relatively mild in nature, it is a global socioeconomic burden [1], [2]. *Rhinoviruses* are also associated with severe respiratory tract illnesses such as pneumonia [3], cystic fibrosis [4], bronchitis [5], chronic obstructive pulmonary disease [6], asthma [7] and whizzing illnesses in infants [8].

*Rhinoviruses* belong to the genus *Enterovirus* and family *Picornaviridae*. *Rhinoviruses* were referred to as *Human rhinoviruses* (HRV) until very recently. The proposal to rename *Human rhinovirus* species as *Rhinovirus,* by removing host name has been approved by the International Committee on Taxonomy of Viruses (ICTV) and is made available online [9]. Accordingly, the term *Rhinoviruses* is being used in place of *Human rhinoviruses* and the three species of *Rhinoviruses* have been re-designated as *Rhinovirus A, -B* and -*C*. However, the serotypes of each of these species are still abbreviated as HRV-A, HRV-B and HRV-C [9]. In view of this, as an example, serotype 2 of *Rhinovirus A* species is referred to as HRV-A2. There are 77 and 25 known serotypes of HRV-A and HRV-B, respectively, based on cross-neutralization assays in cell culture [10], [11]. There are 51 known types of HRV-C. These types are proposed on the basis of molecular phylogeny analysis of VP1 coding region since HRV-C strains are not culturable *in vitro* [12], [13].

*Rhinoviruses* are small, non-enveloped, single-stranded RNA viruses with a positive sense genome of ∼7200 bases. The genome contains a single open reading frame (ORF) which is flanked by 5′ and 3′untranslated regions (UTR). The ORF encodes a single polyprotein that is post-translationally cleaved into four structural proteins (VP1, VP2, VP3 and VP4) and seven non-structural proteins (2A, 2B, 2C, 3A, 3B, 3C and 3D). Sequences of various sub-genomic regions such as 5′UTR [14], VP4/VP2 [15], VP1 [16], 3D polymerase [17] and partial 2A [18] have been used to study evolutionary relatedness of HRV-A, -B and -C strains/isolates.

Serotypes of *Rhinovirus A* and *-B* species have been classified based on their receptor specificity into major and minor receptor group viruses. The major receptor group viruses are those which use intracellular adhesion molecule-1 (ICAM) receptors. The minor receptor group viruses use members of low-density lipoprotein receptor (LDLR) family for entry into the host cell. Twelve serotypes of *Rhinovirus A* (HRV-A1A, -A1B, -A2, -A23, -A25, -A29, -A30, -A31, -A44, -A47, -A49 and -A62) belong to the minor receptor group of viruses. The remaining serotypes of *Rhinovirus A* as well as all the serotypes of *Rhinovirus B* belong to the major receptor group [19], [20].

Genetic diversity of *Rhinoviruses* is attributed to a high mutation rate, which is due to the low fidelity of RNA-dependent RNA polymerase that lacks proof-reading activity. In *Picornaviruses,* RNA polymerase-mediated error rate has been estimated to be between 10^−3^ and 10^−4^ errors/nucleotide/cycle of replication [21]. Additionally, the genetic recombination has also been reported to be a cause behind diversity in *Rhinovirus* species [22], [23]. Recombination is likely to occur through co-infections as well as through successive infections by strains of different species of *Rhinoviruses.* Coinfection of a *Rhinovirus* with other respiratory viruses can also result in recombination [24]. Therefore, use of molecular phylogenetic analysis, a commonly used approach for classification of *Rhinoviruses*, may lead to inappropriate classification, especially in case of recombinant strains [25].

The present study was undertaken to assess the extent of recombination and its impact on genetic diversity in a *Rhinovirus* population. A Bayesian-based population genetics approach [26], which does not depend on phylogenetic analysis, was used to carry out population stratification studies and to identify diversifying lineages within the *Rhinovirus* population. Role of selection pressure was also analyzed to study the contributions of episodic positive selection in *Rhinovirus* evolution and in diversification of *Rhinovirus* population into distinct lineages.

## Materials and Methods

### Compilation and Curation of Dataset

Complete genome sequences of 179 *Rhinovirus* strains were compiled from GenBank [27] and curated using the information of serotype/strain made available by the *Picornaviridae* study group of ICTV [28]. There are 111, 40 and 28 entries respectively for *Rhinovirus A, -B* and -*C* species. The serotype data along with the GenBank accession numbers are provided in Table S1.

### Inference of Population Genetic Structure of Rhinoviruses

*Rhinovirus* population diversity was analyzed using the dataset of 179 complete genome sequences. The major steps in the analysis are multiple sequence alignment (MSA), extraction of parsimony-informative (PI) sites, linkage equilibrium analysis, identification and analysis of genetic structure using Bayesian-based clustering approach and verification of genetic structure using Analysis of Molecular Variance (AMOVA) test. All of these steps, along with the software and various parameters used are described below.

#### Multiple sequence alignment and extraction of parsimony informative sites

MSA of 179 complete genome sequences was carried out using MUSCLE program in MEGA 5.05 [29]. The parsimony informative (PI) sites were then extracted using MEGA and used as an input for STRUCTURE 2.3.3 [26] and LIAN 3.5 [30] programs. A PI site is defined as the site that contains at least two types of nucleotide bases and at least two of them occur with a minimum frequency of two. The gaps were treated as the 5^th^ nucleotide state and ambiguous characters were considered as ‘missing values’. A total of 5689 PI sites were obtained and these were referred to as ‘loci’. The positions of loci in the whole genome alignment were used to generate genetic distance map.

#### Linkage equilibrium analysis

The null hypothesis of linkage equilibrium was tested using LIAN 3.5 program [30]. This program implements Monte Carlo simulations to obtain simulated datasets where loci are resampled without replacement. This program computes a standardized index of association, *I^S^_A_*, which is a measure of the degree of haplotype-wide linkage derived from a dataset and is given by, *I^S^_A_* = [1/(*e* -1)][(*V_D_*/*V_E_*)-1]. Here, *V_D_* is the observed variance of pairwise distances between haplotypes (groups of closely related sequences that apparently share a recent common ancestry) and *V_E_* is the variance expected when all loci are in linkage equilibrium. The term [(*V_D_*/*V_E_*)-1] represents a function of rate of recombination, which equals to zero for being in the state of linkage equilibrium. The number of loci analyzed is represented by *e*.

In addition to *I^S^_A_*, two additional measures of linkage disequilibrium (LD) viz., |D’| and *r^2^* were also computed using DnaSP 5 program [31]. |D’| is the absolute value of the difference between the observed and the expected haplotype frequency in the absence of LD, which is normalised by the maximum (or minimum) possible value of this difference. The squared value of the difference between the observed and the expected haplotype frequency normalised by the variance of the allele frequency, is denoted by *r^2^* [32].

#### Identification and analysis of genetic structure

In order to analyze genetic structure of the *Rhinovirus* population, a Bayesian model-based clustering approach implemented in the STRUCTURE 2.3.3 program [26] was used. The STRUCTURE program provides various ancestry models to deduce population structure and to identify distinct subpopulations each of which is characterized by a set of allele frequencies at every locus. This method attempts to probabilistically assign individuals to populations, while simultaneously estimating allele frequencies in the populations. We used the admixture and linkage models with correlated allele frequencies between populations for the analysis. These models account for individuals having mixed ancestry (potential recombinants) and also help to probabilistically assign such individuals to two or more populations. The linkage model is developed to account for potential linkage between loci and thereby to avoid underestimation or overestimation of the admixed individuals [26], [33].

The admixture model was built using 20,000 burn-in and 40,000 Markov Chain Monte Carlo (MCMC) run lengths. Default values were used for other parameters such as allele frequency parameter (*λ*), Dirichlet parameter for degree of admixture (*α*), etc. The optimum number of clusters (*K_opt_*) represents the number of subpopulations. To determine the *K_opt_*, twenty independent simulation runs were carried out for each value of *K*, ranging from 1 to 13. This analysis led to the calculation of posterior probability of data for a given value of *K* and associated standard deviation, which are used to calculate Δ*K* as suggested in [34]. The plot of *K* versus Δ*K* was finally used to determine the value of *K_opt_*, which is represented by the highest peak in the plot *K_opt_*. Linkage model was built based on 20,000 burn-in, 40,000 MCMC run lengths and 10,000 admixture burn-in length.

### Validation of Genetic Structure Hypothesis

The population genetic structure in *Rhinoviruses* obtained by STRUCTURE 2.3.3 program was validated using *F*_ST_ values (Fixation indices) obtained by applying Analysis of Molecular Variance (AMOVA) test implemented in ARLEQUIN 3.11 software package [35].

### Molecular Phylogeny Analysis of Rhinovirus Population

Molecular phylogeny analysis (MPA) was carried out using the dataset of 179 complete genomes of *Rhinoviruses* using the Neighbor-joining (NJ), the Maximum likelihood (ML) and the Maximum parsimony (MP) methods provided in MEGA [29]. Multiple sequence alignment was obtained using MUSCLE program in MEGA and it was used to generate the phylogenetic trees. MSA file in mega format is provided as Text S1. Bootstrap analysis was carried out by sampling 1000 replicates to estimate essential correctness of resultant phylogenetic tree topology/ies generated using all the three methods. The FigTree 1.2.3 software [36] was used to visualize the phylogenetic trees.

### Recombination Analysis

Complete genome alignment of all 179 *Rhinovirus* strains obtained using MEGA was screened for the presence of potential recombinant sequences and identification of potential parents (major and minor) using various recombination detection methods implemented in the RDP4 program [37]. These methods include RDP [38], GENCONV [39], BOOTSCAN [40], MAXICHI [41], CHIMAERA [42], SiScan [43] and 3SEQ [44]. A stringent p-value cutoff of less than 0.00001 was applied and the multiple comparison correction setting was kept “on” (as default). A sequence is considered as potential recombinant only if it is significantly (with p<0.00001) identified as a recombinant by more than any of the two methods mentioned above. The potential recombinant *Rhinovirus* strains obtained using RDP4 were cross-checked if they were identified as admixed by the STRUCTURE program. The individual is said to be admixed when it has >5% ancestry (or >0.05 membership score) to belong to more than one subpopulation.

### Selection Pressure Analysis

In order to examine potential evidence of selection pressure in the codons of *Rhinovirus*, the potential recombinant sequences identified by RDP4 program were excluded. Thereby the dataset consisting of 133 *Rhinovirus* strains (86 HRV-A, 33 HRV-B and 14 HRV-C) was used for the analysis of selection pressure. Using these entries, 12 separate datasets were prepared which include individual coding sequences of all the 11 proteins (VP1 to VP4, 2A to 2C and 3A to 3D) as well as the complete coding sequence for the polyprotein.

The outcome of positive selection analysis is largely dependent on the choice of the MSA program used. Effect of removing unreliable alignment regions during analysis of positive selection is recently reported [45]. This study indicates that performance of positive selection identification is best for the MSA obtained using program PRANK and worst for MSA obtained using the CLUSTALW program. The MUSCLE and MAFFT programs were found to perform better and were ranked next to the PRANK. Therefore, each of the 12 datasets was independently subjected to the codon alignment using PRANK, MUSCLE and MAFFT programs implemented in the GUIDANCE server [46]. GUIDANCE server is useful to check the quality of the codon alignment because it provides confidence score for entire alignment as well as for each base and column within the alignment. We compared GUIDANCE confidence scores for alignments obtained using PRANK, MUSCLE and MAFFT programs for all the 12 datasets. Since GUIDANCE confidence scores for MUSCLE-based alignments were found to be higher for most of the 12 datasets, alignments derived by MUSCLE were used for selection pressure analysis.

All the 12 datasets were independently analyzed for the evidence of selection pressure (positive or negative) using three different maximum-likelihood methods such as Single Likelihood Ancestor Counting (SLAC) [47], Fixed Effect Likelihoods (FEL) [47] and Internal Fixed Effect Likelihoods (IFEL) [48]. These methods are available at the Datamonkey web-server, which is a part of the HYPHY package [49]–[51]. These three methods basically estimate the ratio of the non-synonymous to synonymous mutations (dN/dS or ω) at every codon in the alignment. The results were analyzed using default setting of *p* = 0.1. The run for identification of the best model was carried out by using automated model selection tool at Datamonkey server. The general time reversible (GTR) model is found to be the best model for *Rhinovirus* datasets and thus subsequently used during selection pressure analysis employing all of the three methods mentioned above.

All the three methods enable detection of the sites that are under pervasive positive selection across all the lineages in the phylogenetic tree. In order to account for the sites that are under episodic positive selection (sites that are positively selected only in a few lineages), a recently developed method, namely, Mixed Effects Model of Evolution (MEME) was used [52]. MEME is available at the Datamonkey server. MEME method integrates both site-to-site rate variation (by employing FEL along the sites) as well as lineage to lineage rate variation (by employing Random-effects likelihood across the branches) to detect episodic diversifying selection. Thus, MEME method is helpful to infer the episodes of diversifying evolution, which may affect only a small subset(s) of lineages even when the majority of the lineages are subjected to the purifying selection.

In order to confirm the evidence of episodic diversifying selection in HRV-A, -B and -C, we have also used Branch-site Random-effects likelihood (BSR) method [53], which is available at the Datamonkey server [49]–[51]. The BSR method helps in identifying nodes/branches in the NJ tree that undergo episodic positive selection. The BSR method provides mapping of the proportion of sites that are under episodic positive selection and also indicates the strength of selection on relevant nodes/branches of phylogenetic tree. Therefore the dataset consisting of complete coding sequences of polyprotein was used to study the effect of episodic selection on diversification of lineages in each of HRV-A, -B and -C.

### Genotype-phenotype Correlation Analysis

The amino acid residues corresponding to the positively selected codons were mapped on the proteins for which three-dimensional structures are available in the Protein Databank (PDB) [54]. Functional implications of such amino acids in antigenicity, drug-resistance or receptor attachment, etc. were also analyzed as the experimental data for antigenic sites [55], [56] and receptor-binding sites [57], [58] for viruses of major and minor receptor groups are available. The hydrophobic drug-binding site within VP1 has already been experimentally characterized [16].

The structures of proteins of HRV-A2 serotype (capsid proteins [PDB ID: 1FPN], 2A protease [PDB ID: 2HRV], 3D polymerase [PDB ID: 1XR6]) were used to represent all the HRV-A minor receptor group serotypes. The structures of proteins of HRV-A16 serotype (capsid proteins [PDB ID: 1AYM], 3D polymerase [PDB ID: 1XR7]) and of HRV-B14 serotype (capsid proteins [PDB ID: 1R09], 3D polymerase [PDB ID: 1XR5]) were respectively used to represent all HRV-A and HRV-B major receptor group serotypes. The three-dimentional structures are visualized and rendered using SwissPDB viewer 3.7 [59].

## Results

In this study, a comprehensive analysis of the complete genome sequences of *Rhinoviruses* has been carried out to understand the role of evolutionary forces such as recombination and selection pressure to study population diversity.

In case of *Rhinoviruses*, role of recombination has been established already [12], [22]–[24], [60]–[62]. The extent of recombination in the population of *Rhinoviruses* was studied using the STRUCTURE program. This program has been developed to infer population structure of haploid, diploid or polyploid organisms [33]. It was initially used to identify various ethnic groups in human populations [63]. Subsequently, the program has been gainfully applied to study genetic structure and admixture in various diploid populations such as those of chimpanzee [64], chicken [65], etc. Its applicability to infer population structure in haploid organism namely *Helicobacter pylori* has been demonstrated for the first time in 2003 [66]. It was later used to analyze haploid populations of organisms such as *Plasmodium falciparum* [67], *Hepatitis B virus* [68], species of the genus *Begomovirus* [69], etc.

Use of the STRUCTURE program to study population of *Rhinovirus* requires validation of the hypothesis that most of the loci are in the state of linkage equilibrium. Therefore, analysis of extent of linkage disequilibrium (LD) in the *Rhinovirus* dataset was carried out.

### Linkage Equilibrium Analysis

In order to test the degree of linkage equilibrium present within *Rhinovirus* genomes, a standardized index of association (*I^S^_A_*) between PI sites across genomes was calculated using LIAN 3.5 program [30]. The value of *I^S^_A_* is expected to be zero in case of free recombination. The *I^S^_A_* value obtained for *Rhinoviruses* was found to be 0.0666 (*p*<10^−4^, 10000 replicates). The value of *I^S^_A_*, though apparently small is found to be significant by the virtue of p-value criteria. It suggests weak evidence of linkage disequilibrium (LD) and indicates nonrandom association of polymorphic loci. In order to confirm low evidence of LD, plots of |D’| and *r^2^* against distance between loci were obtained (data not shown). Average values of |D’| and that of *r^2^* were calculated and found to be 0.5409 and 0.0613, respectively. These values also substantiate weak evidence of LD. These findings together indicate that the polymorphic loci are only weakly correlated and therefore the use of STRUCTURE program to study genetic diversity of *Rhinovirus* population is justified.

### Analysis of *Rhinovirus* Population Structure

**Figure 1:**
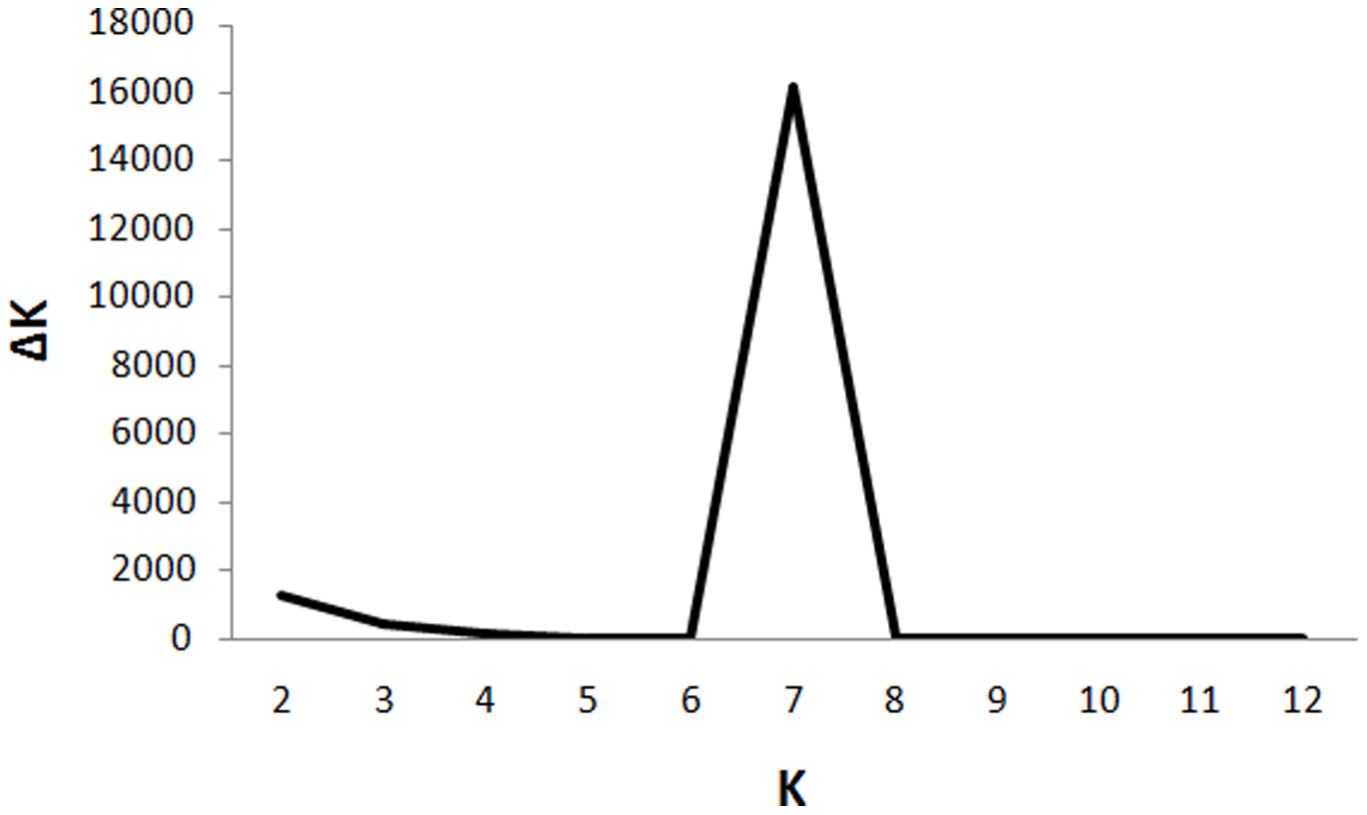
Determination of *K_opt_* using the plot of *K* vs. *ΔK*. *ΔK* represents the rate of change of posterior probability given the number of clusters (*K*). *ΔK* is plotted against *K* to determine optimum number of clusters (*K_opt_*) within *Rhinovirus* population (comprising of HRV-A, -B and -C). The peak at *K* = 7, represents that *K_opt_* is 7 and thus indicate that *Rhinovirus* population is subdivided into seven genetically distinct subpopulations.

**Figure 2:**
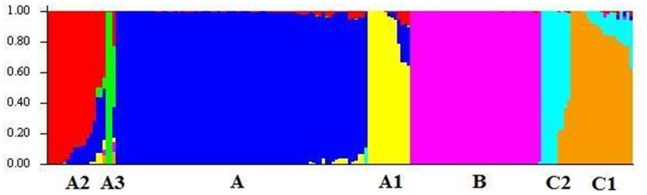
Population structure of *Rhinoviruses* obtained by Bayesian-based clustering approach using admixture model at *K* = 7. HRV-A comprises of four subpopulations viz. -A (blue), -A1 (yellow), -A2 (red), -A3 (green). HRV-B members form a single cluster (magenta) with no further subdivision. HRV-C comprises of two subpopulations viz. C1 (orange) and C2 (cyan). The A1, A2, A3, C1 and C2 subpopulations show presence of several admixed strains. Admixed strains are color coded based on the proportion of membership scores to belong to the respective subpopulations.

In order to study the population structure and to identify recombinants, if any, the admixture model in the STRUCTURE program was used. Twenty independent simulation runs using admixture model (see methods for details) were carried out for *K* = 1 to 13. The *K_opt_* of 7 was determined based on a clear peak of *ΔK* (the rate of change of posterior probability given *K*) obtained using the plot of *K* versus *ΔK* as shown in Figure 1. These observations suggest existence of seven genetically distinct subpopulations of *Rhinoviruses*. Evidence of seven subpopulations was further confirmed by the AMOVA test implemented in the ARLEQUIN 3.11 software [35]. The test statistic, *F*_ST_ value of 0.44 with *p* = 0.0, strongly suggests that the differences among emerging and existing subpopulations of *Rhinoviruses* are statistically significant. Thus, the *K_opt_* of 7 indicates that genetic structure of *Rhinoviruses* consists of three known species viz., HRV-A, -B and -C as well as three additional subpopulations emerging from HRV-A and one additional subpopulation from HRV-C. Schematic representation of subpopulations of *Rhinovirus* population at *K* = 7 (using admixture model) is shown in Figure 2.

Genetic structure of *Rhinovirus* population at varying values of *K* (3 to 7) was also analyzed as there are three known species, however, the *K_opt_* was found to be 7. The STRUCTURE program assigns individuals to one or more subpopulations based on relative membership score that vary from 0 to 1. Membership score of 1 is an indicator of being a member of a cluster, whose members have evolved from a single ancestor. The program also facilitates identification of admixed individuals, which could have evolved due to events such as recombination of strains that belong to different subpopulations. As a result, an admixed individual is assigned with the membership scores to belong to respective subpopulations, which clearly indicate mixed ancestry. In Figure 2, the bar plot depicts various subpopulations by distinct colors. The admixed strains are represented by multiple colors in the bar plot based on their membership scores.

At *K* = 3, all the three known species of *Rhinoviruses* were found to cluster separately as expected. Analysis carried out at *K* = 4, indicated occurrence of admixed strains within *Rhinovirus A* population. This suggests that HRV-A is subdivided into a (potentially) pure subpopulation A and one or more admixture subpopulations. Additionally, a distinct subpopulation is found to be emerging from HRV-A population that consists of 12 serotypes such as HRV-A7, -A20, -A28, -A36, -A51, -A58, -A65, -A71, -A88, -A89, -A102 and -A103. Among these serotypes, HRV-A89 and -A36 show the estimated membership scores of 1 for the 4^th^ cluster while serotypes viz. HRV-A58, -A7, -A88 showed membership scores of >0.70 for the 4^th^ cluster. The estimated membership scores for remaining serotypes were found to be in the range of 0.47 to 0.50, which indicated that these members could also belong to another subpopulation/(s). Hence, analysis of the populations was undertaken at *K* = 5.

Analysis of population stratification at *K* = 5 revealed that HRV-B and -C populations remain undivided and HRV-A strains clustered as three subpopulations viz. subpopulation A and two admixture subpopulations A1 and A2. The A1 subpopulation includes strains of five HRV-A serotypes viz. HRV-A7, -A36, -A58, -A88 and -A89 with membership scores ranging from 0.7 to 1. The A2 subpopulation includes strains of seventeen HRV-A serotypes viz. HRV-A8, -A12, -A20, -A28, -A45, -A46, -A51, -A53, -A65, -A68, -A71, -A78, -A80, -A95, -A101, -A102 and -A103. The membership scores of strains of serotypes belonging to A2 subpopulation were in the range of 0.72 to 0.999, with a few exceptions. These exceptions include serotypes viz. HRV-A8, -A95, -A45, -A12 and -A78 having scores in the range of 0.45 to 0.50. In order to confirm the classification of these five serotypes which have low membership scores, analysis at *K* = 6 was carried out.

At *K* = 6, HRV-C population is subdivided into two subpopulations viz. C1 and C2. Majority of the HRV-C strains belonged to the C1 subpopulation. The C1 subpopulation includes nineteen HRV-C strains showing membership scores in the range of 0.63 to 1. While the C2 subpopulation consists of nine HRV-C strains showing membership scores in the range of 0.56 to 1. The members of the C2 subpopulation include two HRV-C6 strains (strain 026 [GenBank: EF582387] and strain HRV-C06_p1031_sR2724_2009 [GenBank: JN990702]), two HRV-C3 strains (QPM [GenBank: EF186077] and HRV-C03_p1280_s6359_1999 [GenBank: JN798567]), isolate LZ651 [GenBank: JF317016], HRV-C1 strain NAT001 [GenBank: EF077279], HRV-C10 strain QCE [GenBank: GQ323774], HRV-C7 strain NY074 [GenBank: DQ875932] and HRV-C43 strain namely HRV-C43_p1154_sR1124_2009 [GenBank: JX074056]. Membership scores indicate no discrepancy in the assignment of HRV-C strains to their respective C1 or C2 subpopulations, however, assignment of HRV-A strains viz. HRV-A8, -A95, -A45 and -A12 was not resolved at *K* = 6. Hence analysis of clustering pattern at *K* = 7 was carried out.

At *K* = 7, the subpopulation A2 is observed to further subdivide into another subpopulation named A3. This subpopulation includes three HRV-A serotypes viz. HRV-A8, -A95 and -A45. The two serotypes viz. HRV-A8 and -A95 show membership scores of 0.989 and 0.987, respectively, to belong to the subpopulation A3 while HRV-A45 show admixture from subpopulations A3 (membership score 0.43), A1 (membership score 0.22) and pure A (membership score 0.20). Thus, the highest membership score suggests that HRV-A45 belongs to the A3 subpopulation. However, HRV-A12 and -A78 showed highest membership scores to belong to A2 viz. 0.41 and 0.46, respectively. This suggests that HRV-A12 and -A78 are members of A2 subpopulation. Figure 2 clearly shows subdivision of HRV-A population into four subpopulations such as A, A1, A2 and A3. Similarly, HRV-C population is subdivided into subpopulations C1 and C2 while the *Rhinovirus B* population shows no further subdivisions. Similar analysis at *K* = 8 produced inconsistent clustering during repeated runs, which further confirmed that the *Rhinovirus* population is subdivided only in seven distinct subpopulations as also observed by plotting *K* versus *ΔK* (Figure 1).

Subsequently, analysis employing linkage model at *K* = 7 was carried out to confirm assignment of admixed strains to respective subpopulations. Linkage model also validated clustering of *Rhinovirus* strains into seven distinct genetic subpopulations (A, A1, A2, A3, B, C1 and C2). The bar plot obtained by the linkage model is given in the Figure S1. The results obtained using linkage model substantiate that the admixed strains were correctly identified by the admixture model except in case of few strains belonging to the subpopulation A2 and C2. The linkage model provided evidence of inter-subpopulation admixture in case of five HRV-C strains (HRV-C3 [GenBank: EF186077], HRV-C1 [GenBank: EF077279], HRV-C10 [GenBank: GQ323774], HRV-C7 [GenBank: DQ875932] and HRV-C43 [GenBank: JX074056]). These strains show admixture membership scores to belong to the subpopulations of HRV-A species, especially A2, in addition to the C1 and C2 subpopulations. In case of subpopulation A2, linkage model uniquely helped to provide evidence of admixture amongst the members of subpopulation A2.

### Sublevel Clustering in *Rhinovirus A*

**Figure 3:**
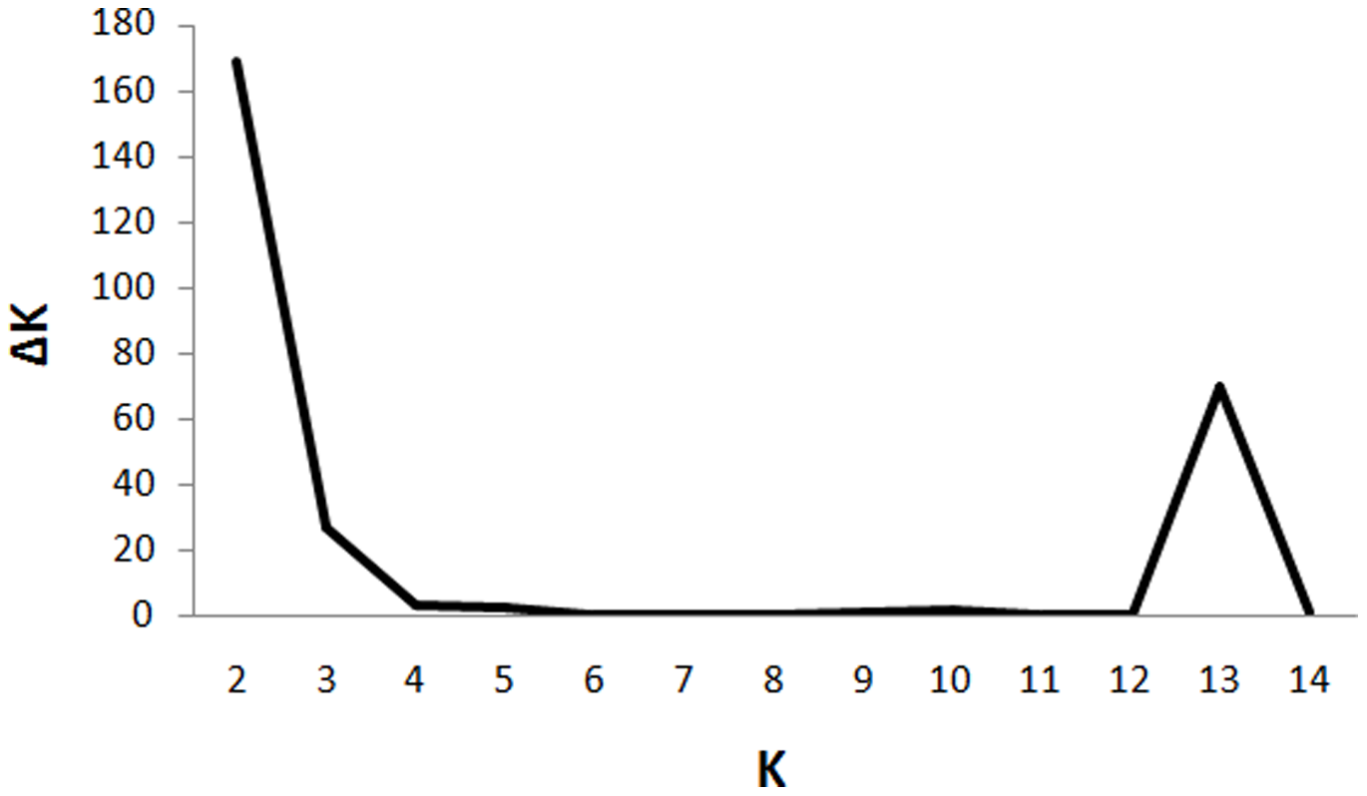
Sublevel clustering of HRV-A: The plot of *K* vs. *ΔK* obtained for HRV-A strains. *ΔK* represents the rate of change of posterior probability given the number of clusters (*K*). *ΔK* is plotted against *K* to determine optimum number of clusters (*K_opt_*) within *Rhinovirus A* population. A major peak at *K* = 2 and a minor peak at *K* = 13 is observed. It suggests that *Rhinovirus A* population primarily divides into two major groups. The minor peak at *K* = 13 indicates that *Rhinovirus A* population is further subdivided into 13 minor subpopulations.

In order to validate the subdivision of *Rhinovirus A* population into four subpopulations (A, -A1, -A2, -A3) and to study extent of intra-species admixture, the dataset consisting of 111 genomes of HRV-A strains were subjected to independent sublevel clustering using the STRUCTURE program. The plot of *K* versus *ΔK* indicates highest value of *K* at 2 followed by a clear peak at *K* = 13 (Figure 3). The clustering obtained at *K* = 2 confirmed that the *Rhinovirus A* population primarily divides into two subgroups. The first subgroup consists of the members of the subpopulation A while the second subgroup comprises of three subpopulations A1, A2 and A3.

Presence of substructure within *Rhinovirus A* population at *K* = 13 is validated using AMOVA test in ARLEQUIN software. *F*_ST_ value of 0.42 (*p* = 0) obtained at *K* = 13 supports existence of thirteen sublevel subpopulations within *Rhinovirus A* population. The members belonging to thirteen subclusters are listed in Table S2. Interestingly, this result also suggests presence of A1, A2 and A3 as genetically distinct subpopulations. The subpopulation A2 is subdivided into two subclusters and members of subpopulation A are divided into nine distinct subclusters. No further subdivision of A1 and A3 subpopulations was observed. The grouping of these subclusters is also in agreement with the topology of phylogenetic tree obtained using NJ-based method (described below).

### Sublevel Clustering in *Rhinovirus C*

**Figure 4:**
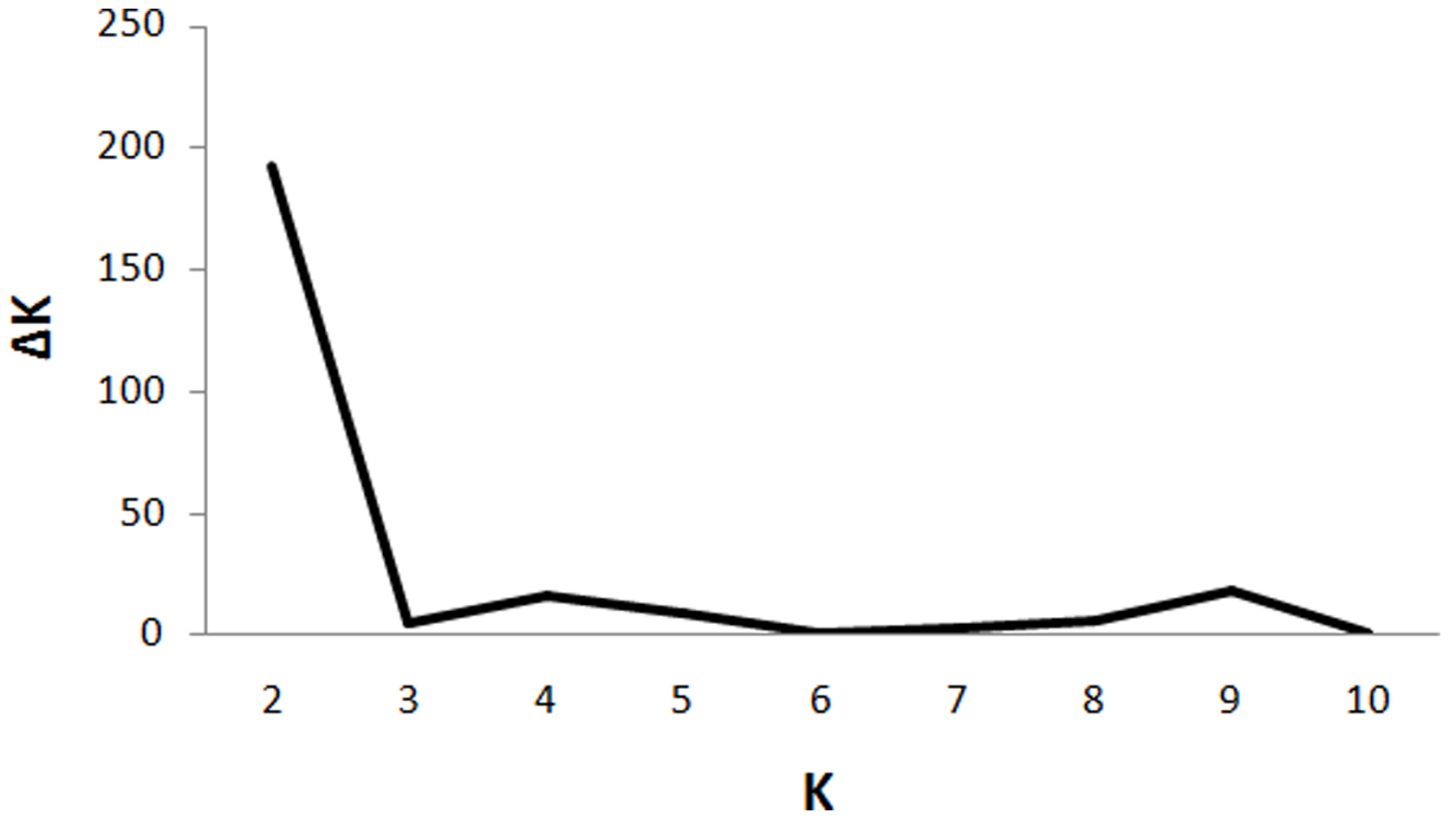
Sublevel clustering of HRV-C: The plot of *K* vs. *ΔK* obtained for HRV-C strains. *ΔK* represents the rate of change of posterior probability given the number of clusters (*K*). *ΔK* is plotted against *K* to determine optimum number of clusters (*K_opt_*) within *Rhinovirus C* population. A major peak at *K* = 2 and two minor peaks at 4 and 9 are observed. The major peak at *K* = 2 suggests that *Rhinovirus C* population primarily divided into two major groups (which correspond to the C1 and C2 subpopulations as mentioned in the text). The peak at *K* = 9, represents optimum number of minor subpopulations within HRV-C based on significant *F*_ST_ value of 0.47.

The plot of *K* versus *ΔK* (Figure 4) suggests an initial peak at *K* = 2 followed by two clear peaks at 4 and 9. The *F*_ST_ value of 0.32 (*p* = 0) was obtained for *K* = 4, which is lower than the cutoff for the significant *F*_ST_ value_._ The *F*_ST_ of 0.47 (*p* = 0) obtained for *K* = 9 is statistically significant and provides evidence of sublevel genetic structure within HRV-C. This observation suggests presence of nine sublevel subpopulations in HRV-C. The C1 subpopulation is subdivided into seven sublevel subpopulations while the C2 subpopulation is divided into two sublevel subpopulations. It also implies that two subpopulations in HRV-C could be further diversified into multiple subpopulations (Table S3). However, genomic data of additional HRV-C types would be necessary to substantiate further subdivision of HRV-C.

### Molecular Phylogeny Analysis of *Rhinovirus* Population

**Figure 5:**
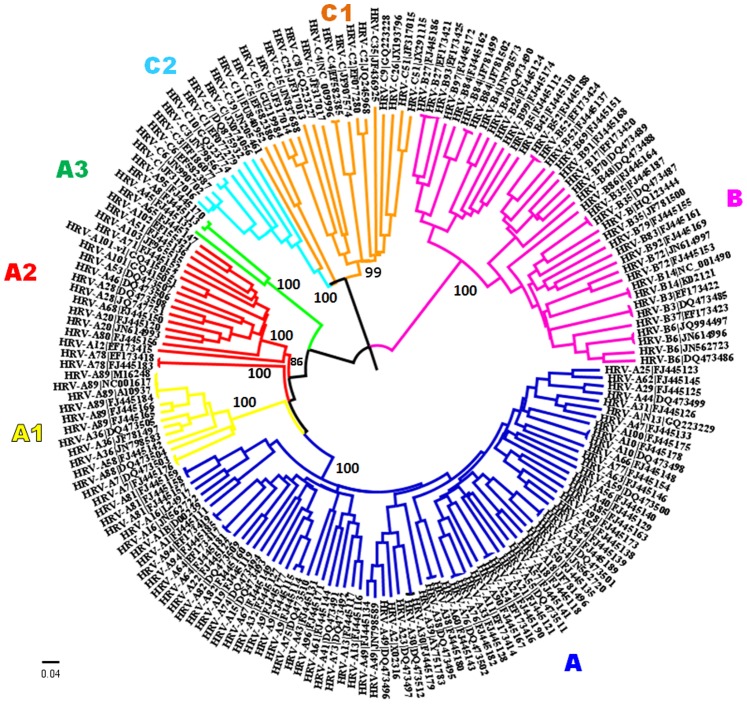
Phylogenetic tree of *Rhinoviruses* obtained using Neighbor-joining method in MEGA 5.05. Complete genome sequence data with 1000 bootstrap replicates was used. The operational taxonomic unit (OTU) label consists of two parts divided by pipe (‘|’) character. The first part (before ‘|’) indicates species-serotype and second part constitute GenBank accession number of the associated entry. The branches in the tree are color coded as per the seven subpopulations obtained using STRUCTURE program [Subpopulation A: blue, A1: yellow, A2: red, A3: green, B: magenta, C1: orange, C2: cyan].

Phylogenetic analysis was carried out using Neighbor-joining (NJ), Maximum likelihood (ML) and Maximum parsimony (MP) methods and trees are shown as Figures 5, S2 and S3 respectively. The phylogenetic trees generated using all the three methods revealed that there are seven major clusters, which correspond to the seven subpopulations (A, A1, A2, A3, B, C1 and C2), obtained by the STRUCTURE program. Further, topologies of all the three trees divide HRV-A species into four subpopulations and HRV-C species into two subpopulations. The order of clustering of evolutionarily related serotypes within every subpopulation was also found to be similar in all the three trees barring the recombinant strains. As can be seen from Figure 5, the order of clustering of species in the NJ tree was HRV-A (innermost), HRV-C (intermediate) and HRV-B (outermost), which is in accordance with the whole genome-based NJ tree, published earlier [22]. However, the order of clustering of species in the trees generated using MP and ML methods was HRV-A (innermost), HRV-B (intermediate) and HRV-C (outermost). The interchange in the order of HRV-B and HRV-C clusters could be attributed to the underlying models of respective phylogenetic methods. Therefore, reconstruction of phylogeny using NJ, MP and ML methods was also carried out by including complete genomes of the type species of the genus *Enterovirus* as an outgroup. The outgroup included three serotypes of the type species *Enterovirus C* such as *Human coxsackievirus A13* (GenBank: AF499637), *Human coxsackievirus A21* (GenBank: AF546702) and *Human poliovirus 1* (GenBank: V01149). The resultant MP and ML trees (not shown) depicted similar order of species clustering (HRV-A, -C and -B) as observed in both the NJ trees, without outgroup (Figure 5) and with outgroup (tree not shown).

As mentioned above, differential placement was observed for recombinant/admixed strains such as HRV-A78, HRV-A12, HRV-A46, HRV-A80 and HRV-C39 in all the three trees (Figures 5, S2 and S3). The molecular phylogenetic methods fail to resolve the placement of recombinant strains [25]. For example, two strains of HRV-A78 serotype, which belong to the subpopulation A2 were observed to share an immediate evolutionary ancestor with the members of subpopulation A1 and A rather than A2, in both, NJ and ML trees (Figure 5 and S2). The population stratification results obtained using STRUCTURE program (using admixture model), suggest that HRV-A78 serotype is admixed with membership scores of 0.46 and 0.34 for A2 and pure A subpopulations, respectively. The membership scores show that HRV-A78 strains are the members of A2 subpopulation, however, further sublevel clustering analysis of only HRV-A population using STRUCTURE program indicates that HRV-A78 strains form a distinct lineage, which might evolve independently over the period of time.

The HRV-C39 strain, which belongs to the C1 subpopulation (according to the STRUCTURE program), clusters with the members of C2 subpopulation in all the three trees (Figures 5, S2 and S3). The membership scores of HRV-C39 suggest that it is an admixed strain having major membership to belong to C1 subpopulation and minor membership to belong to C2 subpopulation. Thus, it is advisable to interpret the results of phylogenetic trees in the light of population genetics analysis and vice versa. The phylogenetic trees obtained using NJ and ML methods for proteome dataset (trees not shown) also corroborates with the NJ tree obtained using complete genomes (Figure 5).

### Recombination Analysis Using RDP4 & STRUCTURE Programs

Extent of recombination in *Rhinovirus* genomes was analyzed using p-value cutoff of <0.00001 in RDP4 program [40]. The analysis revealed that there are a total of 46 potential recombinant strains (Table S4), out of which 23 recombinant strains were previously reported [22]. The admixed strains identified using the STRUCTURE program were further analyzed using the RDP4 program. Both these programs suggest that the majority of the recombinants were the members of A2, C1 and C2 subpopulations and there was a good agreement between results obtained by both the programs. For example, results obtained by RDP4 program suggest that strains belonging to HRV-A101 serotype are recombinants having HRV-A65 (which belongs to subpopulation A2) as a major parent and HRV-A78 (which belongs to subpopulation A) as a minor parent. Similarly, the STRUCTURE program identifies HRV-A101 strains as admixed (at *K_opt_* = 7, linkage model) and assigns the membership scores of 0.81 and 0.16 to belong to subpopulations A2 and A respectively. Thus, while the STRUCTURE program suggests potential contribution of respective subpopulations to form admixed individuals, the RDP4 program helps to identify the extent of recombination from respective strains. The admixture(s) between two distinct subpopulations were correctly captured by the STRUCTURE program at *K* = 7 using linkage model. Furthermore, subpopulation A in itself constitutes potential recombinants having both the parents belonging to the subpopulation A itself. Such “within-subpopulation admixture” could also be correctly interpreted using the HRV-A sublevel clustering results obtained at *K* = 13. For example, analysis using the RDP4 program suggests that, one member of subpopulation A, namely, HRV-A38 is a potential recombinant of HRV-A9 (major parent) and HRV-A98 (minor parent), both of the parents belonging to subpopulation A. Therefore, HRV-A38 is shown (by the STRUCTURE program) to have membership of ∼1 to belong to subpopulation A implying no admixture/recombination. On the other hand, sublevel clustering of HRV-A, at *K* = 13 revealed that HRV-A38 is admixed strain having membership scores of 0.164 and 0.154 to belong to sublevel HRV-A clusters represented by strains HRV-A9 and HRV-A98, respectively.

The analysis carried out using RDP4 program also substantiated the evidence of recombination in two known minor receptor group viruses (HRV-A31, HRV-A47) and one potential minor receptor group strain (HRV-A N13). All the three viruses mentioned above have the HRV-A54 as a major parent (a member of major receptor group) and HRV-A25 as a minor parent (a member of minor receptor group). As can be seen in Figure 5, the minor group viruses form a single monophyletic cluster, which in turn clusters with their parent (HRV-A54) that belongs to major receptor group viruses.

The subpopulation A3 consists of only three strains viz. HRV-A8, -A95 and -A45. According to the STRUCTURE program, HRV-A45 is an admixed strain while HRV-A8 and -A95 are not admixed. The HRV-A45 had membership scores for A2 (0.213), A (0.191) and A3 (0.448) subpopulations, respectively while HRV-A8 and -A95 have membership score of 1 to belong to A3 subpopulation. The analysis carried out using RDP4 program at *p*<0.00001 reveals that HRV-A45 is not a recombinant whereas evidence of recombination in HRV-A45 was obtained at relaxed p-value (<0.001). It appears that data of additional strains of A3 subpopulation would be required to confirm extent of recombination events, if any.

Analysis of recombination in HRV-C population using RDP4 program revealed that eight out of nine strains of C2 subpopulation were recombinants (Table S4). Out of these eight recombinant strains, seven were shown to have a minor parent belonging to HRV-A species, viz. HRV-A-101-v1 [GenBank: GQ415052]. The recombination events were observed to have occurred in either the 5′UTR region or in the 5′UTR-polyprotein junction. The major parent of these recombinant strains however could not be ascertained using RDP4 program. The STRUCTURE program using linkage model (at *K* = 7), however, provided significant evidence of inter-species admixture in five strains (out of seven strains identified by RDP4 program) belonging to the C2 subpopulation viz. HRV-C3 [GenBank: EF186077], HRV-C1 [GenBank: EF077279], HRV-C10 [GenBank: GQ323774], HRV-C7 [GenBank: DQ875932] and HRV-C43 [GenBank: JX074056]. The outcome of the STRCUTURE program further indicates that these strains also show membership scores to belong to both C1 and A2 subpopulations, in addition to the major subpopulation C2. It implies that these recombinant strains are likely to have a major parent belonging to the C2 subpopulation. Thus the C2 subpopulation/lineage is likely to be derived as a result of inter-species admixture between HRV-A and HRV-C strains.

### Selection Pressure Analysis: Evidence of Pervasive Positive Selection

A total of 12 datasets were analyzed for the potential evidence of positive selection pressure. Only one codon viz. 881 was found to be positively selected when the dataset of complete coding sequences of polyprotein of all the three species was analyzed. This codon corresponds to the amino acid position 267 in VP1 protein and is known to be a part of the antigenic site B [56]. This site is a part of neutralizing epitope in minor receptor group viruses. The strains of minor receptor group of viruses have amino acid viz. aspartic acid (D) at position 267, which is replaced by glycine (G) or serine (S), in major receptor group viruses. Similarly, an independent analysis of dataset consisting of VP1 also revealed the evidence of positive selection at the same codon, which corresponds to residue 267 of VP1. For this codon, the difference (dN-dS) of 29.23 (*p* = 0.007) and dN/dS ratio of 2.720 (*p* = 0.0257) were obtained using SLAC and IFEL methods, respectively. However, no significant evidence (p<0.1) of pervasive positive selection was obtained for the remaining gene datasets. Overall, majority of the codon sites in all the 11 genes were significantly found to be under purifying selection.

### Selection Pressure Analysis: Evidence of Episodic Positive Selection

A total of 11 datasets corresponding to every gene were analyzed separately using MEME method [52]. Significant evidence of episodic positive selection (p<0.05) was obtained in a total of 29 codons. These codons are found within the coding regions of only three structural (VP1, VP2, VP3) and three non-structural (2A, 2C and 3D) proteins. The detail results are given in Table 1.

**Table 1:** The codons under episodic diversifying selection identified using MEME method are reported.

Codon	Gene	α	β−	Pr[β = β−]	β +	Pr[β = β+]	p-value	q-value
80	VP1	0.818827	0.026499	0.963851	5.81875	0.03615	0.030001	1
86	VP1	0.59647	0.169525	0.956509	25.5085	0.043492	0.045927	1
88	VP1	2.5947	0.281093	0.953641	169.285	0.046359	0.034481	1
89	VP1	1.93481	0.074462	0.955312	34.5748	0.044688	0.011377	1
91	VP1	1.13654	0.164593	0.958643	30.3555	0.041357	0.028799	1
95	VP1	0.360492	0.055921	0.822938	4.73397	0.177062	0.005586	1
200	VP1	1.2864	0.16781	0.966253	45.8939	0.033747	0.039125	1
228	VP1	0.783171	0.167934	0.944388	13.3426	0.055612	0.013985	1
268	VP1	0.7984	0.065732	0.808318	4.52442	0.191682	0.037699	1
271	VP1	0.684146	0.226078	0.927104	111.598	0.072896	0.041559	1
278	VP1	0.938812	0.194511	0.767753	7.50235	0.232247	0.046182	1
285	VP1	0.509856	2.55E-09	0.848437	104.566	0.151563	0.008591	1
104	VP2	0.590612	0.023302	0.964667	20.4757	0.035333	0.011858	0.806322
235	VP2	0.60462	0.036361	0.840895	7.75313	0.159105	0.000188	0.051198
262	VP2	0.491395	2.46E-09	0.845732	3.00207	0.154268	0.002772	0.251284
263	VP2	0.640999	0.06049	0.880133	16.0186	0.119867	0.002535	0.344701
171	VP3	0.338841	1.69E-09	0.970198	13.553	0.029802	0.000445	0.053816
180	VP3	0.507421	0.00024	0.943632	2.65874	0.056368	0.047268	1
234	VP3	0.800654	0.019767	0.904056	3.60533	0.095944	0.02723	1
236	VP3	0.297792	1.49E-09	0.840911	2.54098	0.159089	2.74E-05	0.006622
2	2A	1.31761	0.009664	0.987332	166.458	0.012668	0.003557	0.263229
44	2A	0.121099	0.019851	0.752722	1.50307	0.247278	6.75E-05	0.009995
74	2A	1.20588	0.060225	0.983008	154.611	0.016992	0.014075	0.694348
141	2A	1.45877	7.29E-09	0.974452	166.458	0.025548	0.018007	0.666249
72	2C	3.84614	0.015398	0.97934	228.631	0.02066	0.012802	1
88	2C	1.0663	5.33E-09	0.978839	28.1586	0.021161	0.036158	1
189	2C	0.028546	0.002887	0.982178	3.18819	0.017822	0.005165	1
258	3D	0.926377	0	0.960963	33.3659	0.039038	0.000965	0.445619
347	3D	0.884788	0.102909	0.867767	5.27739	0.132233	0.034829	1

A total of 29 codon sites within *Rhinovirus* genome are significantly (p<0.05) found to be under episodic diversifying selection using MEME method [52]. The codon sites are numbered according to the HRV-A2 genome (GenBank: X02316). Majority of these sites are found to be within three structural (VP1, VP2 and VP3) while few sites also within non-structural (2A, 2C and 3D) proteins. This table reports the distribution of synonymous (α) and non-synonymous (β) substitution rates over sites as inferred by MEME. β- represents the maximum likelihood estimate (MLE) of the non-synonymous rate for the branch class with β≤α. Pr[β = β-] represents the MLE of the proportion of sites evolving at β- β+represents the MLE of the unconstrained β non-synonymous rate. Pr[β = β+] represents The MLE of the proportion of sites evolving at β+. The p-value is derived using a mixture of χ^2^ distributions, and q-values are obtained using Sime’s procedure, which controls the false discovery rate under the strict neutral null (likely to be conservative).

A total of 12 codons within VP1 were found to be under significant episodic positive selection. Further mapping with the phenotypic properties revealed that out of the 12 codons, 8 codons were associated either with antigenicity or with LDLR-receptor binding site in minor receptor group viruses. The codons 86, 91 and 95 (numbering according to HRV-A2 [GenBank: X02316]) are known to be part of antigenic site A [55] while the codons 268 and 278 are the part of known antigenic site B [56] of the minor receptor group viruses. Similarly, the codons 88, 89 and 228 are known to occur in the LDLR footprint region [58]. The amino acid coded by codon 80 is reported to be within 0.04 Å of the ICAM-binding site [70].

In case of VP2, four codons viz. 104, 235, 262 and 263 were found to be under significant episodic selection. The amino acid (serine) coded by the codon 235 precedes the known antigenic residue (alanine) that belongs to antigenic site C in the minor receptor group viruses [56].

The four codons viz. 171, 180, 234 and 236 in VP3 gene were found to be under significant episodic positive selection. The codon 180 in HRV-A2 (minor receptor group virus) corresponds to the codon 178 in HRV-B14 (major receptor group virus). In HRV-B14, proline at the 178 position is known to be the part of ICAM-binding site [70]. Similarly, the codons 234 and 236 in HRV-A2 corresponds to the amino acid positions 233 and 235 in HRV-B14 and are observed to be in close proximity of ICAM-binding site [70].

The 2A gene consists of four codons viz. 2, 44, 74 and 141 under significant episodic positive selection. The codon 44 (GTA) corresponds to amino acid residue valine (V) in the protein 2A of HRV-A2 serotype. Three-dimensional structure of 2A protein is available only for HRV-A2 serotype (PDB ID: 2HRV) [71]. It has been reported that residues 40–52 form an inter-domain loop between N- and C-terminal domains. The codon 74 corresponds to the amino acid serine (S). This residue is a part of beta II2 strand of the beta-barrel forming the C-terminal domain. The codon 141 (GAA) is a part of the C-terminal end for which three-dimensional structure is not yet solved [71].

In case of 2C gene, three codons viz. 72, 88 and 189 were identified to be under significant episodic positive selection. In *Picornaviruses,* 2C protein is one of the highly conserved proteins and is multifunctional. It is known to possess NTPase and ATPase activities. The NTPase domain contains three conserved motifs (A, B and C) which are characteristic of superfamily III of the NTPase/helicase proteins [72]. The codon 189 encodes amino acid residue which is adjacent to the known B-motif (formed by residues at positions 176–187) within NTPase domain. However, its structural mapping could not be analyzed due to the unavailability of its crystal structure.

In 3D polymerase gene, two codons were identified to be under significant episodic positive selection viz. 258 and 347. Both of these codons encode amino acid residues which occur within functionally important domains. The codon 258 encodes for serine (S), which belongs to the inner fingers of the fingers domain and is observed to be within the loop region joining the two helices [73]. The codon 347 encode for lysine (K), which belongs to the motif D, which is one of the highly conserved known motifs for oligonucleotide polymerases. The motif D (residues 338–355) is present within a helix (α16) in the palm domain (residues 291–373) [73].

### Episodic Diversifying Selection in Newly Emerging Lineages

In order to check the evidence of episodic positive selection leading to the diversification of HRV-A, -B and -C into distinct lineages (as obtained by the STRUCTURE program), the Branch-site REL (BSR) analysis was performed. The three datasets consisting of complete polyprotein coding sequences of HRV-A, -B and -C were analyzed separately. This method identifies the nodes/branches in the trees that are under episodic diversifying selection pressure. For *Rhinovirus A*, BSR analysis provided significant evidence (p<0.05) of episodic diversifying selection on 16 nodes/branches of the tree. The BSR method strongly indicates episodic diversifying selection acting on the node 7 (p<0.0001) which leads to the lineages A1, A2 and A3. Among all the 16 branches, the branches under the node 7 showed the higher strength of episodic diversifying selection (Figure S4). The tree in the Figure S4 was generated by excluding the serotypes HRV-A53 and HRV-A45, which represents the subpopulations A2 and A3 respectively. Inclusion of these serotypes in this analysis also supports the evidence of episodic positive selection at node 7 but the proportion of sites under selection in these serotypes are so high that it affects the representation of the strength of episodic diversifying selection for other serotypes, as evident from resultant tree topologies (tree not shown).

In case of HRV-B, BSR method strongly supports episodic diversifying selection acting on the node 4 (p<0.0001) which leads to the lineage comprising of the drug-resistant serotypes (see Figure S5). In case of HRV-C, no significant episodic diversifying selection was observed using the BSR method.

## Discussion

A high mutation rate and intra-species recombination are the eminent evolutionary forces introducing genetic variability within various species belonging to the genus *Enterovirus* [74] to which *Rhinoviruses* belong. In general, RNA viruses are excellent systems to study evolution under the theoretical framework of population genetics because of comparatively higher mutation rate, short generation time, large population sizes and relatively smaller size of genomes [75].

### Evidence of Population Structure and Role of Recombination in the Emergence of New Lineages in *Rhinoviruses*

A number of attempts have been made to analyze genetic diversity of *Rhinovirus*es and emerging taxonomic lineages have been documented [12], [22], [60], [76]. These studies were carried out using either subgenomic region(s) or limited data on complete genome sequences, especially for newly identified HRV-C strains. A comprehensive complete genome-based analysis was carried out to analyze population diversity of *Rhinoviruses* and to understand the evolution of diversifying lineages in HRV-A, -B and -C species.

Sublevel structure analysis of HRV-A population (at *K* = 2) clearly distinguished members of subpopulation A from that of three additional subpopulations viz., A1, A2 and A3. This indicates that subpopulations A1, A2 and A3 initially shared common allele frequency at specific loci and diverged later. Population stratification analyses suggest that A1 members showed the clearest signal of divergence (at *K* = 4) followed by divergence of A2 (at *K* = 5) and A3 members (at *K* = 7) at subsequent values of *K*. The members of A3 continue to cluster with A2 members (till *K* = 6) and thereby suggest that A3 shared common allele frequency with A2 and diverged subsequently as evident by the population diversification analysis at *K_opt_* = 7. The cluster A3 consisting of HRV-A8, -A45 and -A95 has been identified as a distinct clade. These strains were proposed earlier as potentially the fourth species of *Rhinoviruses* (HRV-D) based on the complete genomic analyses [22]. However, separation of A1 and A2 subclusters prior to separation of A3 strongly emphasizes existence and consideration of A1, A2 and A3 subpopulations as independently evolving lineages of HRV-A. Phylogenetic studies that were based on sequences of VP1 and 3D-pol proteins also support this subdivision of members of HRV-A into four genetically distinct lineages [23].

Population genetic structure analysis of *Rhinoviruses* reported here provides strong evidence for existence of seven genetically distinct subpopulations using admixture as well as linkage model. Admixture model tend to ignore physical linkage between loci and at times may under- or over-estimate the proportion of admixed individuals [26], [33]. Hence, linkage model, which accounts for potential linkage, is applied to refine membership scores of admixed individuals [33]. In case of HRV-A population, intra-species recombination was observed especially in A, A2 and A3 subpopulations. In these subpopulations, the assignments of almost all the admixed strains to belong to the respective subpopulations were observed to remain unchanged using both, admixture and linkage models. This observation supports evidence of intra-species recombination in these strains irrespective of any potential occurrence of the physical linkage between loci. The low values of linkage measures (|D’|, *r^2^* and *I^S^_A_*) also substantiate only weak evidence of linkage and supports for recombination in origin and evolution of *Rhinovirus* strains.

Recombination analyses (using both STRUCTURE and RDP4 programs) suggest that majority of the A2 members are recombinants. HRV-A101 is derived through recombination between A2 members HRV-A65 and -A78 and has been proposed as a new lineage within HRV-A [62]. Another A2 member, HRV-A28 is also reported to be a recombinant of A2 members such as HRV-A68 and -A20. Similarly, HRV-A46 is also a recombinant of HRV-A53 and -A80 (all three belong to the A2 subpopulation) [22]. Presence of highest number of recombinants, the emergence of newly proposed A3 members from A2 and subdivision of A2 subpopulation into two distinct subclusters observed using population stratification analysis supports the hypothesis that the members of the A2 subcluster are more likely to undergo frequent recombination events leading to evolution of new strains/lineages of HRV-A.

In case of HRV-C population, linkage model was found to be advantageous over the admixture model in detecting the inter-species admixture events in several HRV-C strains belonging to the C2 subpopulation. The complete genome based population structure analysis along with the results obtained using the RDP4 program provide strong evidence of emergence of C2 subpopulation as a distinct lineage due to inter-species recombination between the HRV-A and HRV-C, mainly in the 5′UTR region. It corroborates well with the earlier observation that the inter-species recombination between the strains of HRV-A and HRV-C exists [60]. Furthermore, 5′-UTR-polyprotein junction has been experimentally found to have potential to undergo recombination and is reported to be involved in the inter-species recombination among as well as between the members of *Rhinovirus*es and *Human enterovirus* (HEVs) species [77]. A recent study reports presence of three species-like clusters (Cα, Cβ and Cγ) within HRV-C based on analysis of the dataset consisting of six proteins which are conserved across family *Picornaviridae* [76]. There are four strains of proposed Cα cluster viz., C026, NY-074, NAT001 and QPM, representing the types HRV-C6, -C7, -C1 and -C3 respectively. Our analysis demonstrates that these strains belong to the C2 subpopulation. The strains that are proposed to form Cβ and Cγ sub-species are grouped under C1 subpopulation in our analysis. Thus, the population stratification studies based on complete genomes indicate that there are only two subpopulations of HRV-C which further could be subdivided into nine sublevel subpopulations. Additional genomic data and analysis is however required to conclusively arrive at subspecies organization of the HRV-C.

### Role of Episodic Positive Selection in *Rhinovirus* Evolution and Genotype-phenotype Correlations

Evidence of genetic structure and diversifying lineages in *Rhinoviruses* led us to undertake a study to understand role of lineage specific episodic positive selection pressure, if any, using MEME method [52]. MEME method has also been used to study the emergence of genetically divergent lineages in *Simian Immunodeficiency Virus* (SIV) and *Human Immunodeficiency Virus* (HIV), *Human Respiratory Syncytial Virus* (RSV) and *Foot and-mouth disease virus* (FMDV) [78]–[80].

Substantial evidence for episodic diversifying selection was obtained and it appears to be responsible for diversification of HRV-A population into three additional lineages (A1, A2 and A3). Episodic positive selection is found to have operated both on regions coding for structural and non-structural proteins. In case of structural proteins, most of the sites under episodic diversifying selection were found to be associated with phenotypic properties such as antigenicity and/or receptor-binding specificity in HRV-A and -B. Most of the antigenic sites that were mapped happened to be characteristics of minor receptor group viruses. These results also corroborate with the previously reported observation that minor receptor group viruses undergo positive destabilizing selection while major group viruses undergo positive stabilizing selection [81].

A recent study based on modeling of capsid of type HRV-C15 reports major structural alterations in the loop regions of VP1 [82]. In HRV-C, the length of VP1 protein is shorter by 21 and 35 amino acid residues as compared to HRV-A and -B respectively. These deletions lead to structural alterations by means of loss of 5-fold plateau in HRV-C virus particle, as against HRV-A and -B [82]. As a result, neutralizing immunization (Nim) sites namely Nim-1A (residues 91 and 95), Nim-1B (residues 82 and 85) are subjected to increased selection pressure in HRV-C.

Different receptor usage by various genera of *Picornaviridae* as well as other virus families has been proposed to be a useful mechanism to escape antibody neutralization. Coevolution of receptor usage and antigenicity has also been observed in various picornaviruses such as poliovirus, FMDV as well as other RNA viruses [83]. Consequently, the viral capsid region(s) in general and the receptor attachment sites in particular are reported to be subjected to strong selection pressure [83], [84], implying possible involvement of independent receptor(s) by HRV-C. It is interesting to note that two independent approaches involving selection pressure analysis reported in this paper and mapping of epitope data on three-dimensional structure [82] help to correlate genotype with phenotype. Analysis of population diversity alongwith genotype-phenotype correlation studies is expected to play increasingly important role not only in designing new vaccines and drugs but also to in explaining emergence of drug resistance amongst viral subpopulations.

## Conclusions

The population stratification studies based on complete genomes using the STRUCTURE program clearly demonstrates diversification of *Rhinovirus* population into seven distinct lineages. This study establishes that HRV-A and -C have further diversified into four and two distinct subpopulations, respectively. Besides role of intra-species recombination, an evidence of episodic positive selection in evolution of newly emerging lineages within HRV-A is also reported. Evolution of HRV-C seems to be driven by intra-species as well as inter-species recombination with HRV-A. This study adds new understanding of origin and evolution of emerging lineages within *Rhinovirus* population. It also furthers our knowledge about *Rhinovirus* taxonomic diversity and may lead to bring out their subspecies organization more clearly.

## Supporting Information

Figure S1 **Population structure of** ***Rhinoviruses*** **obtained by Bayesian-based approach, using linkage model at** ***K*** **= 7.** HRV-A comprises of four subpopulations, namely, A (yellow), A2 (orange), A3 (green). HRV-B members form a single cluster (blue) with no further subdivision. HRV-C comprises of two subpopulations, namely, C1 (magenta) and C2 (Cyan). The A1, A2, A3, C1 and C2 subpopulations show the admixed strains. They are color-coded based on the proportion of membership scores with respective subpopulations.
(TIF)

Figure S2 **Phylogenetic tree of** ***Rhinoviruses*** **obtained using Maximum likelihood method in MEGA 5.05.** Complete genome sequence data with 1000 bootstrap replicates was used. The operational taxonomic unit (OTU) label consists of two parts divided by pipe (‘|’) character. The first part (before ‘|’) indicates species-serotype and second part constitute GenBank accession number of the associated entry. The branches in the tree are color coded as per the seven subpopulations obtained using STRUCTURE program [Subpopulation A: blue, A1: yellow, A2: red, A3: green, B: magenta, C1: orange, C2: cyan].
(TIF)

Figure S3 **Phylogenetic tree of** ***Rhinoviruses*** **obtained using Maximum parsimony method in MEGA 5.05.** Complete genome sequence data with 1000 bootstrap replicates was used. The operational taxonomic unit (OTU) label consists of two parts divided by pipe (‘|’) character. The first part (before ‘|’) indicates species-serotype and second part constitute GenBank accession number of the associated entry. The branches in the tree are color coded as per the seven subpopulations obtained using STRUCTURE program [Subpopulation A: blue, A1: yellow, A2: red, A3: green, B: magenta, C1: orange, C2: cyan]. Note: For the ease of readability of bootstrap and OTU labels, tree is shown in rectangular representation.
(TIF)

Figure S4 **Evidence of episodic diversifying selection in** ***Rhinovirus A*** **obtained using Branch-site REL method.**
(PDF)

Figure S5 **Evidence of episodic diversifying selection in Rhinovirus B obtained using Branch-site REL method.**
(PDF)

Table S1 **Complete genome sequence dataset of 179 Rhinoviruses with GenBank accession numbers used in the study.**
(DOC)

Table S2 **Sublevel clustering of Rhinovirus A and -C obtained at K = 9, using the STRUCTURE program.**
(DOC)

Table S3 **Sublevel clustering of Rhinovirus C obtained at K = 9, using the STRUCTURE program.**
(DOC)

Table S4 **Recombinant strains identified using RDP4 program.**
(DOC)

Text S1 **Multiple sequence alignment of 179 complete genomes of** ***Rhinoviruses*** **, obtained using MUSCLE program in MEGA 5.05.** The alignment was used for the phylogenetic tree reconstruction using various methods (Neighbor-joining, Maximum likelihood and Maximum parsimony) in MEGA 5.05.
(MEG)
