# Supplementary figures and images for: Population Structure and Evolution of Rhinoviruses
Source: PLoS One. 2014 Feb 19;9(2):e88981. doi: 10.1371/journal.pone.0088981 (PMC3929619; doi:10.1371/journal.pone.0088981)

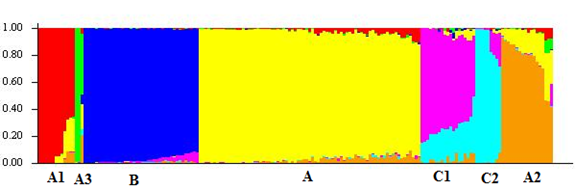

Supplement: Figure S1 — Population structure of Rhinoviruses obtained by Bayesian-based approach, using linkage model at K = 7. HRV-A comprises of four subpopulations, namely, A (yellow), A2 (orange), A3 (green). HRV-B members form a single cluster (blue) with no further subdivision. HRV-C comprises of two subpopulations, namely, C1 (magenta) and C2 (Cyan). The A1, A2, A3, C1 and C2 subpopulations show the admixed strains. They are color-coded based on the proportion of membership scores with respective subpopulations. (TIF) [file pone.0088981.s001.tif]

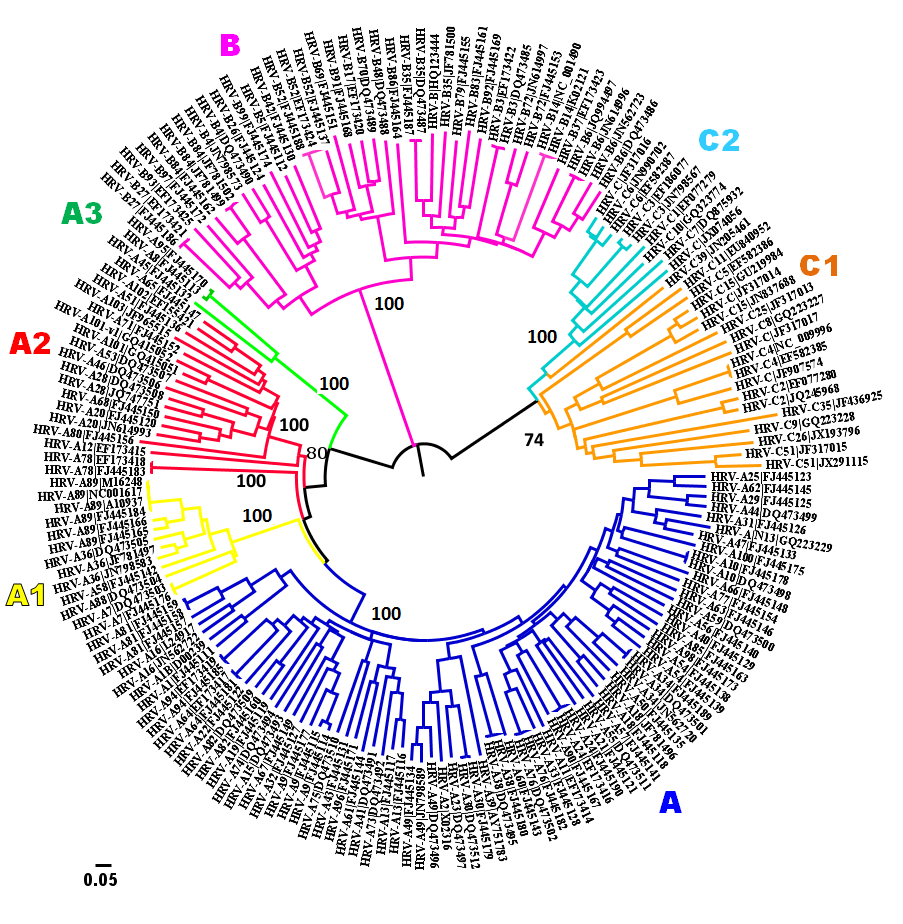

Supplement: Figure S2 — Phylogenetic tree of Rhinoviruses obtained using Maximum likelihood method in MEGA 5.05. Complete genome sequence data with 1000 bootstrap replicates was used. The operational taxonomic unit (OTU) label consists of two parts divided by pipe (‘|’) character. The first part (before ‘|’) indicates species-serotype and second part constitute GenBank accession number of the associated entry. The branches in the tree are color coded as per the seven subpopulations obtained using STRUCTURE program [Subpopulation A: blue, A1: yellow, A2: red, A3: green, B: magenta, C1: orange, C2: cyan]. (TIF) [file pone.0088981.s002.tif]

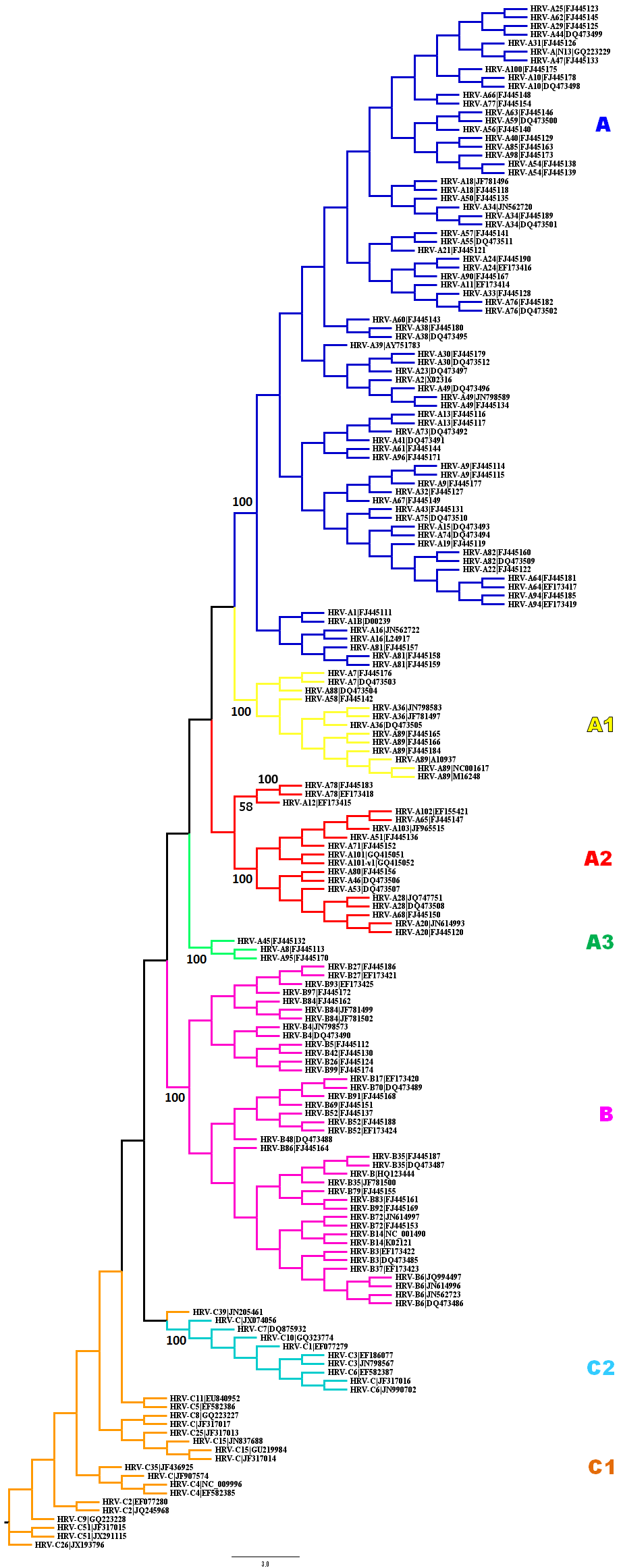

Supplement: Figure S3 — Phylogenetic tree of Rhinoviruses obtained using Maximum parsimony method in MEGA 5.05. Complete genome sequence data with 1000 bootstrap replicates was used. The operational taxonomic unit (OTU) label consists of two parts divided by pipe (‘|’) character. The first part (before ‘|’) indicates species-serotype and second part constitute GenBank accession number of the associated entry. The branches in the tree are color coded as per the seven subpopulations obtained using STRUCTURE program [Subpopulation A: blue, A1: yellow, A2: red, A3: green, B: magenta, C1: orange, C2: cyan]. Note: For the ease of readability of bootstrap and OTU labels, tree is shown in rectangular representation. (TIF) [file pone.0088981.s003.tif]

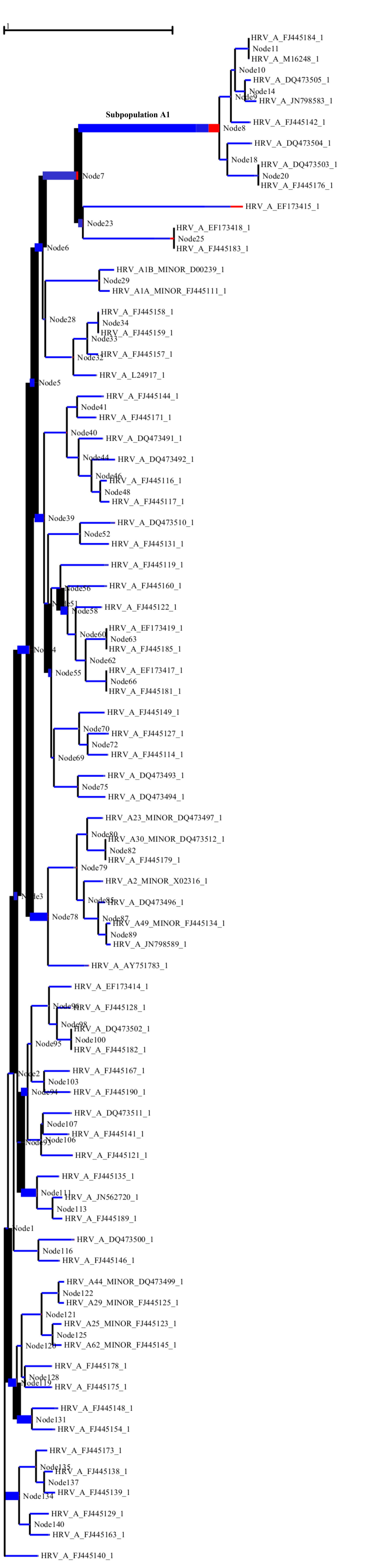

Supplement: Figure S4 — Evidence of episodic diversifying selection in Rhinovirus A obtained using Branch-site REL method. (PDF) [file pone.0088981.s004.pdf]

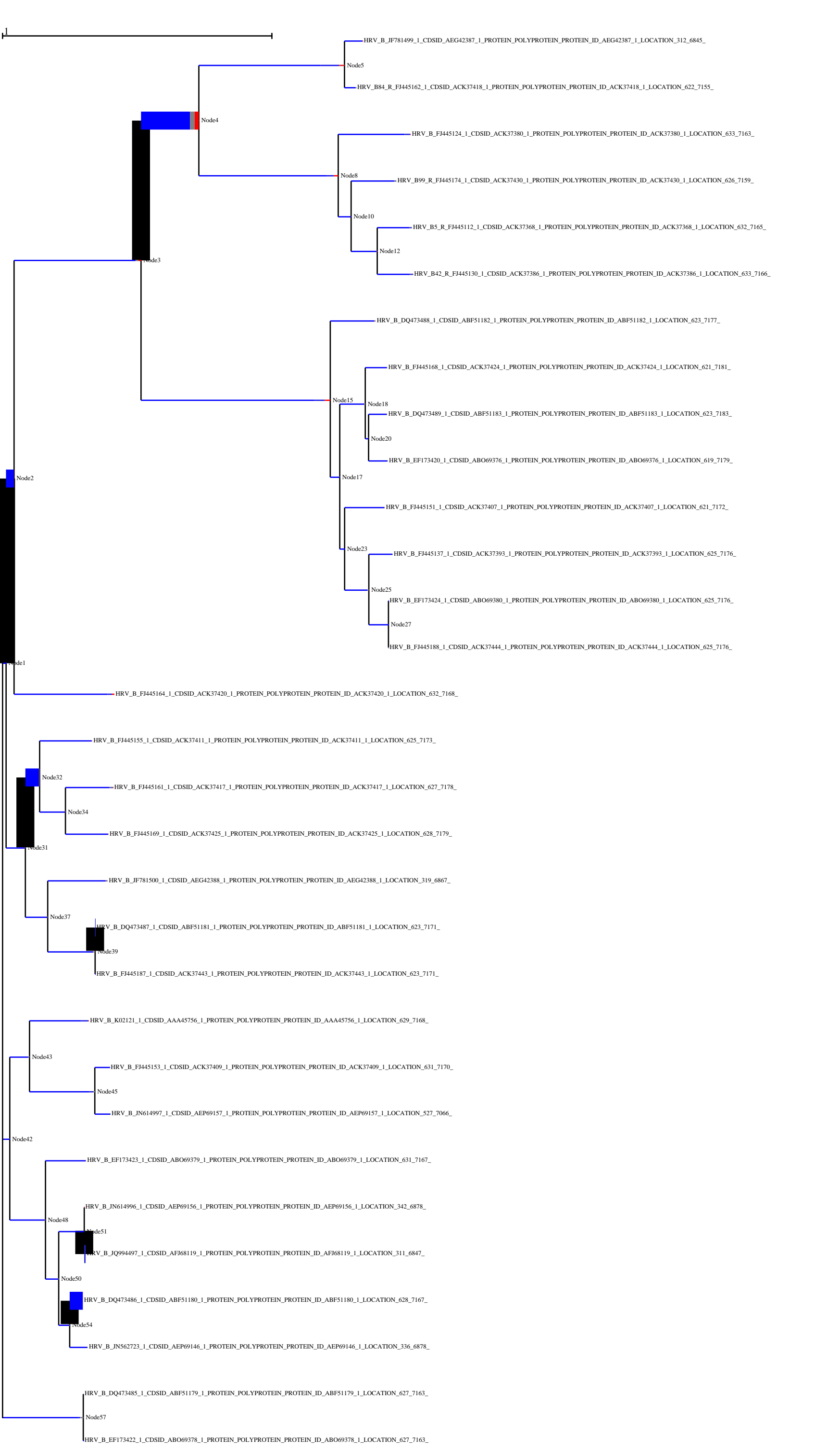

Supplement: Figure S5 — Evidence of episodic diversifying selection in Rhinovirus B obtained using Branch-site REL method. (PDF) [file pone.0088981.s005.pdf]
