# Supplementary material for: Population Structure and Evolution of Rhinoviruses
Source: PLoS One. 2014 Feb 19;9(2):e88981. doi: 10.1371/journal.pone.0088981 (PMC3929619; doi:10.1371/journal.pone.0088981)
Supplement: Table S1 — Complete genome sequence dataset of 179 Rhinoviruses with GenBank accession numbers used in the study. (DOC) [file pone.0088981.s006.doc]

**Table S1: complete genome sequence dataset of 179 *Rhinoviruses* with GenBank accession numbers used in the study.**

| **Sr. no.** | **HRV serotype/strain** | **Genbank Accession Number** |
| --- | --- | --- |
| 1 | HRV-A28 | JQ747751 |
| 2 | HRV-A36 | JN798583 |
| 3 | HRV-A49 | JN798589 |
| 4 | HRV-A34 | JN562720 |
| 5 | HRV-A16 | JN562722 |
| 6 | HRV-A20 | JN614993 |
| 7 | HRV-A102 | EF155421 |
| 8 | HRV-A103 | JF965515 |
| 9 | HRV-A18 | JF781496 |
| 10 | HRV-A36 | JF781497 |
| 11 | HRV-A101 | GQ415051 |
| 12 | HRV-A101-v1 | GQ415052 |
| 13 | HRV-A-N13 | GQ223229 |
| 14 | HRV-A1A | FJ445111 |
| 15 | HRV-A8 | FJ445113 |
| 16 | HRV-A9 | FJ445114 |
| 17 | HRV-A9 | FJ445115 |
| 18 | HRV-A13 | FJ445116 |
| 19 | HRV-A13 | FJ445117 |
| 20 | HRV-A18 | FJ445118 |
| 21 | HRV-A19 | FJ445119 |
| 22 | HRV-A20 | FJ445120 |
| 23 | HRV-A21 | FJ445121 |
| 24 | HRV-A22 | FJ445122 |
| 25 | HRV-A25 | FJ445123 |
| 26 | HRV-A29 | FJ445125 |
| 27 | HRV-A31 | FJ445126 |
| 28 | HRV-A32 | FJ445127 |
| 29 | HRV-A33 | FJ445128 |
| 30 | HRV-A40 | FJ445129 |
| 31 | HRV-A43 | FJ445131 |
| 32 | HRV-A45 | FJ445132 |
| 33 | HRV-A47 | FJ445133 |
| 34 | HRV-A49 | FJ445134 |
| 35 | HRV-A50 | FJ445135 |
| 36 | HRV-A51 | FJ445136 |
| 37 | HRV-A54 | FJ445138 |
| 38 | HRV-A54 | FJ445139 |
| 39 | HRV-A56 | FJ445140 |
| 40 | HRV-A57 | FJ445141 |
| 41 | HRV-A58 | FJ445142 |
| 42 | HRV-A60 | FJ445143 |
| 43 | HRV-A61 | FJ445144 |
| 44 | HRV-A62 | FJ445145 |
| 45 | HRV-A63 | FJ445146 |
| 46 | HRV-A65 | FJ445147 |
| 47 | HRV-A66 | FJ445148 |
| 48 | HRV-A67 | FJ445149 |
| 49 | HRV-A68 | FJ445150 |
| 50 | HRV-A71 | FJ445152 |
| 51 | HRV-A77 | FJ445154 |
| 52 | HRV-A80 | FJ445156 |
| 53 | HRV-A81 | FJ445157 |
| 54 | HRV-A81 | FJ445158 |
| 55 | HRV-A81 | FJ445159 |
| 56 | HRV-A82 | FJ445160 |
| 57 | HRV-A85 | FJ445163 |
| 58 | HRV-A89 | FJ445165 |
| 59 | HRV-A89 | FJ445166 |
| 60 | HRV-A90 | FJ445167 |
| 61 | HRV-A95 | FJ445170 |
| 62 | HRV-A96 | FJ445171 |
| 63 | HRV-A98 | FJ445173 |
| 64 | HRV-A100 | FJ445175 |
| 65 | HRV-A7 | FJ445176 |
| 66 | HRV-A9 | FJ445177 |
| 67 | HRV-A10 | FJ445178 |
| 68 | HRV-A30 | FJ445179 |
| 69 | HRV-A38 | FJ445180 |
| 70 | HRV-A64 | FJ445181 |
| 71 | HRV-A76 | FJ445182 |
| 72 | HRV-A78 | FJ445183 |
| 73 | HRV-A89 | FJ445184 |
| 74 | HRV-A94 | FJ445185 |
| 75 | HRV-A34 | FJ445189 |
| 76 | HRV-A24 | FJ445190 |
| 77 | HRV-A11 | EF173414 |
| 78 | HRV-A12 | EF173415 |
| 79 | HRV-A24 | EF173416 |
| 80 | HRV-A64 | EF173417 |
| 81 | HRV-A78 | EF173418 |
| 82 | HRV-A94 | EF173419 |
| 83 | HRV-A41 | DQ473491 |
| 84 | HRV-A73 | DQ473492 |
| 85 | HRV-A15 | DQ473493 |
| 86 | HRV-A74 | DQ473494 |
| 87 | HRV-A38 | DQ473495 |
| 88 | HRV-A49 | DQ473496 |
| 89 | HRV-A23 | DQ473497 |
| 90 | HRV-A10 | DQ473498 |
| 91 | HRV-A44 | DQ473499 |
| 92 | HRV-A59 | DQ473500 |
| 93 | HRV-A34 | DQ473501 |
| 94 | HRV-A76 | DQ473502 |
| 95 | HRV-A7 | DQ473503 |
| 96 | HRV-A88 | DQ473504 |
| 97 | HRV-A36 | DQ473505 |
| 98 | HRV-A46 | DQ473506 |
| 99 | HRV-A53 | DQ473507 |
| 100 | HRV-A28 | DQ473508 |
| 101 | HRV-A82 | DQ473509 |
| 102 | HRV-A75 | DQ473510 |
| 103 | HRV-A55 | DQ473511 |
| 104 | HRV-A30 | DQ473512 |
| 105 | HRV-A39 | AY751783 |
| 106 | HRV-A16 | L24917 |
| 107 | HRV-A89 | A10937 |
| 108 | HRV-A89 | NC001617 |
| 109 | HRV-A1B | D00239 |
| 110 | HRV-A89 | M16248 |
| 111 | HRV-A2 | X02316 |
| 112 | HRV-B (strain CU211) | HQ123444 |
| 113 | HRV-B6 | JQ994497 |
| 114 | HRV-B4 | JN798573 |
| 115 | HRV-B6 | JN562723 |
| 116 | HRV-B6 | JN614996 |
| 117 | HRV-B72 | JN614997 |
| 118 | HRV-B84 | JF781499 |
| 119 | HRV-B35 | JF781500 |
| 120 | HRV-B84 | JF781502 |
| 121 | HRV-B5 | FJ445112 |
| 122 | HRV-B26 | FJ445124 |
| 123 | HRV-B42 | FJ445130 |
| 124 | HRV-B52 | FJ445137 |
| 125 | HRV-B69 | FJ445151 |
| 126 | HRV-B72 | FJ445153 |
| 127 | HRV-B79 | FJ445155 |
| 128 | HRV-B83 | FJ445161 |
| 129 | HRV-B84 | FJ445162 |
| 130 | HRV-B86 | FJ445164 |
| 131 | HRV-B91 | FJ445168 |
| 132 | HRV-B92 | FJ445169 |
| 133 | HRV-B97 | FJ445172 |
| 134 | HRV-B99 | FJ445174 |
| 135 | HRV-B27 | FJ445186 |
| 136 | HRV-B35 | FJ445187 |
| 137 | HRV-B52 | FJ445188 |
| 138 | HRV-B17 | EF173420 |
| 139 | HRV-B27 | EF173421 |
| 140 | HRV-B3 | EF173422 |
| 141 | HRV-B37 | EF173423 |
| 142 | HRV-B52 | EF173424 |
| 143 | HRV-B93 | EF173425 |
| 144 | HRV-B3 | DQ473485 |
| 145 | HRV-B6 | DQ473486 |
| 146 | HRV-B35 | DQ473487 |
| 147 | HRV-B48 | DQ473488 |
| 148 | HRV-B70 | DQ473489 |
| 149 | HRV-B4 | DQ473490 |
| 150 | HRV-B14 | NC001490 |
| 151 | HRV-B14 | K02121 |
| 152 | HRV-C15 | GU219984 |
| 153 | HRV-C11 | EU840952 |
| 154 | HRV-C10 | GQ323774 |
| 155 | HRV-C9 | GQ223228 |
| 156 | HRV-C8 | GQ223227 |
| 157 | HRV-C4 | NC_009996 |
| 158 | HRV-C6 | EF582387 |
| 159 | HRV-C5 | EF582386 |
| 160 | HRV-C4 | EF582385 |
| 161 | HRV-C3 | EF186077 |
| 162 | HRV-C1 | EF077279 |
| 163 | HRV-C2 | EF077280 |
| 164 | HRV-C7 | DQ875932 |
| 165 | HRV-C (strain HRV-C49_p1102_sR889_2008) | JF907574 |
| 166 | HRV-C25 | JF317013 |
| 167 | HRV-C (isolate LZY79) | JF317014 |
| 168 | HRV-C51 | JF317015 |
| 169 | HRV-C (isolate LZ651) | JF317016 |
| 170 | HRV-C (isolate LZY101) | JF317017 |
| 171 | HRV-C51 | JX291115 |
| 172 | HRV-C39 | JN205461 |
| 173 | HRV-C43 | JX074056 |
| 174 | HRV-C2 | JQ245968 |
| 175 | HRV-C06 | JN990702 |
| 176 | HRV-C15 | JN837688 |
| 177 | HRV-C3 | JN798567 |
| 178 | HRV-C35 | JF436925 |
| 179 | HRV-C26 | JX193796 |
