# Supplementary material for: Population Structure and Evolution of Rhinoviruses
Source: PLoS One. 2014 Feb 19;9(2):e88981. doi: 10.1371/journal.pone.0088981 (PMC3929619; doi:10.1371/journal.pone.0088981)
Supplement: Table S2 — Sublevel clustering of Rhinovirus A and -C obtained at K = 9, using the STRUCTURE program. (DOC) [file pone.0088981.s007.doc]

**Table S2: Sublevel clustering of *Rhinovirus A* obtained at K=13, using STRUCTURE program.** The subdivision of HRV-A serotypes into 13 distinct clusters is shown.

| **Subpopulation** | **Subcluster number** | **HRV- A serotypes** |
| --- | --- | --- |
| A | 1 | HRV-A16 , -A81, -A1A, -A1B |
|  | 2 | HRV-A9, -A32,- A67, -A74, -A15 |
|  | 3 | HRV-A13, -A41, -A73, -A61, -A96 |
|  | 4 | HRV-A30,-A49,-A23,-A2,-A39 |
|  | 5 | HRV- A10,-A100,-A66,-A56,-A44,-A29,-A47,-A62,-A (strain N13),-A77 |
|  | 6 | HRV-A31,-A25,-A54,-A98,-A85,-A40,-A63,-A59 |
|  | 7 | HRV-A90, -A18, -A57, -A21, -A55, -A38, -A60, -A34, -A76, -A18, -A24, -A33, -A50, -A11 |
|  | 8 | HRV-A64, -A94, -A22, -A82, -A19, -A43, -A75 |
|  | 9 | HRV-A78 |
| A1 | 10 | HRV-A89,-A36,-A58,-A7,-A88 |
| A2 | 11 | HRV-A65,-A102,-A51,-A103,-A71,-A101,-A101-v1,-A12 |
|  | 12 | HRV-A28,-A68,-A53,-A46,-A80,A-20 |
| A3 | 13 | HRV-A8,-A45,-A95 |
