# Supplementary material for: Population Structure and Evolution of Rhinoviruses
Source: PLoS One. 2014 Feb 19;9(2):e88981. doi: 10.1371/journal.pone.0088981 (PMC3929619; doi:10.1371/journal.pone.0088981)
Supplement: Table S3 — Sublevel clustering of Rhinovirus C obtained at K = 9, using the STRUCTURE program. (DOC) [file pone.0088981.s008.doc]

**Table S3**: **Sublevel clustering of *Rhinovirus C* obtained at K=9, using STRUCTURE program.** The subdivision of strains of HRV-C into 9 distinct clusters is shown. C1 subpopulation subdivided into 7 subclusters whereas C2 subpopulation is subdivided into 2 subclusters.

| **Subpopulation** | **Subcluster number** | **HRV- C types/strains** |
| --- | --- | --- |
| C1 | 1 | HRV-C4 [NC_009996, EF582385, JF907574] |
|  | 2 | HRV-C15 [GU219984, JN837688], HRV-C (isolate LZY79) [JF317014] |
|  | 3 | HRV-C51 [JF317015, JX291115] |
|  | 4 | HRV-C9 [JF436925],-C35 [JF436925], -C26 [JX193796] |
|  | 5 | HRV-C11 [EU840952], C5 [EF582386] |
|  | 6 | HRV-C2 [EF077280, JQ245968] |
|  | 7 | HRV-C (isolate LZY101) [JF317017], C25 [JF317013], C8 [GQ223227] , C39 [JN205461] |
| C2 | 8 | HRV-C43 [JX074056], -C3 [JN798567] |
|  | 9 | HRV-C6 [EF582387, JN990702], HRV-C3 [EF186077], -C (isolate LZ651) [JF317016], -C1 [EF077279], -C10 [GQ323774], -C7 [DQ875932] |
