# Supplementary material for: Population Structure and Evolution of Rhinoviruses
Source: PLoS One. 2014 Feb 19;9(2):e88981. doi: 10.1371/journal.pone.0088981 (PMC3929619; doi:10.1371/journal.pone.0088981)
Supplement: Table S4 — Recombinant strains identified using RDP4 program. (DOC) [file pone.0088981.s009.doc]

**Table S4. Recombinant strains identified using RDP4 program.**

| Sr.no. | Recombinant  [GenBank Accession no] | Major  parent | Minor  parent | Breakpoint  start | Breakpoint  end | Methods: RGBMCSQ | Lowest P-value |
| --- | --- | --- | --- | --- | --- | --- | --- |
| Subpopulation A2 | | | | | | | |
| 1 | HRV-A28[JQ747751] | A65 | A21 | 20 | 519 | RG**B**MCS | 2.00E-23 |
|  |  | A68 | Unknown | 1039 | 4106 | MC**S** | 4.90E-11 |
| 2 | HRV-A20[JN614993] | A51 | A24 | 0 | 602 | RG**B**MCS | 4.38E-30 |
| 3 | HRV-A101[GQ415051] | A65 | A78 | 11 | 674 | R**B**MCS | 4.42E-15 |
| 4 | HRV-A101-v1[GQ415052] | A53 | A78 | 11 | 506 | R**B**MCS | 4.42E-15 |
| 5 | HRV-A20[FJ445120] | A51 | A24 | 0 | 691 | **R**GBMCS | 4.38E-30 |
| 6 | HRV-A68[FJ445150] | A51 | A24 | 12 | 692 | RG**B**MCS | 4.38E-30 |
| 7 | HRV-A71[FJ445152] | A28 | Unknown | 28 | 752 | R**B**S | 3.69E-10 |
| 8 | HRV-A80[FJ445156] | A65 | A21 | 11 | 789 | RG**B**MCS | 2.00E-24 |
| 9 | HRV-A28[DQ473508] | A68 | Unknown | 1025 | 4379 | MC**S** | 4.92E-11 |
| 10 | HRV-A46[DQ473506] | A53 | A80 | 153 | 3278 | RBM**C**SQ | 8.71E-25 |
| 11 | HRV-A102[EF155421] | A28 | Unknown | 10 | 724 | R**B**S | 3.69E-10 |
| 12 | HRV-A103[JF965515] | A28 | Unknown | 28 | 823 | R**B**S | 3.69E-10 |
| 13 | HRV-A51[FJ445136] | A28 | Unknown | 31 | 642 | R**B**S | 3.69E-10 |
| 14 | HRV-A65[FJ445147] | A28 | Unknown | 28 | 750 | RB**S** | 3.69E-10 |
| Subpopulation A3 | | | | | | | |
| 15 | HRV-A8[FJ445113] | A45 | A21 | 28 | 609 | RG**B**MCS | 1.13E-22 |
| 16 | HRV-A95[FJ445170] | A45 | A21 | 134 | 611 | RG**B**MCS | 1.13E-22 |
| Subpopulation A | | | | | | | |
| 17 | HRV-A31[FJ445126] | A54 | A25 | 852 | 4792 | MC**S** | 4.74E-14 |
| 18 | HRV-A47[FJ445133] | A54 | A25 | 955 | 4830 | MC**S** | 4.74E-14 |
| 19 | HRV-A60[FJ445143] | A9 | A47 | 5165 | 6376 | RGBMC**S** | 7.39E-19 |
|  |  | A9 | A98 | 6639 | 7138 | RB**S** | 8.66E-09 |
| 20 | HRV-A18[JF781496] | A44 | A50 | 838 | 3270 | MC**S** | 1.70E-18 |
| 21 | HRV-A18[FJ445118] | A44 | A50 | 901 | 3411 | MC**S** | 1.70E-18 |
| 22 | HRV-A-N13[GQ223229] | A54 | A25 | 975 | 4463 | M**C**S | 4.48E-14 |
| 23 | HRV-A38[FJ445180] | A9 | A47 | 5157 | 6601 | RGBMC**S** | 7.39E-19 |
| 24 | HRV-A38[DQ473495] | A9 | A47 | 5157 | 6601 | RGBMC**S** | 7.39E-19 |
|  |  | A9 | A98 | 6375 | 7136 | RB**S** | 6.39E-07 |
| 25 | HRV-A82[DQ473509] | A82 | A88 | 0 | 179 | R**G**BMC | 1.42E-34 |
| Subpopulation B | | | | | | | |
| 26 | HRV-B(CU211)[HQ123444] | B35 | B69 | 788 | 1507 | **R**GBMCSQ | 3.75E-38 |
|  |  | B35 | B35 | 4535 | 5134 | R**G**BMCS | 8.97E-51 |
|  |  | B35 | B35 | 5724 | 6537 | RG**B**MCS | 6.08E-60 |
| 27 | HRV-B4[JN798573] | Unknown | B42 | 26 | 387 | RG**B**MCS | 4.36E-19 |
| 28 | HRV-B97[FJ445172] | B42 | B37 | 105 | 690 | RG**B**S | 1.01E-10 |
| 29 | HRV-B27[FJ445186] | B42 | B37 | 49 | 491 | RG**B**S | 1.01E-10 |
| 30 | HRV-B27[EF173421] | B42 | B37 | 49 | 491 | RG**B**S | 1.01E-10 |
| 31 | HRV-B93[EF173425] | B42 | B37 | 49 | 491 | RG**B**S | 1.01E-10 |
| 32 | HRV-B4[DQ473490] | B42 | B97 | 0 | 690 | RG**B**MCS | 4.36E-19 |
| Subpopulation C1 | | | | | | | |
| 33 | HRV-C9[GQ223228] | C2 | Unknown | 0 | 627 | RG**B**MS | 4.93E-20 |
|  |  | Unknown | C8 | 6668 | 7060 | R**G**BS | 2.80E-67 |
| 34 | HRV-C2[EF077280] | C35 | C11 | 0 | 517 | R**B**MCS | 3.49E-13 |
| 35 | HRV-C39[JN205461] | Unknown | HRV-A101-v1 | 0 | 601 | RBMCS | 2.75E-13 |
| 36 | HRV-C2[JQ245968] | C35 | C11 | 1 | 606 | RBMCS | 1.76E-15 |
| 37 | HRV-C51[JX291115] | HRV-C51 | HRV-C15 | 839 | 958 | RGBCS | 1.24E-14 |
|  |  | HRV-C51 | HRV-C15 | 3825 | 3997 | RG**B** | 1.41E-15 |
|  |  | HRV-C51 | HRV-C15 | 4058 | 4111 | **R**GB | 7.37E-16 |
|  |  | HRV-C51 | HRV-C15 | 4233 | 4353 | **R**GBS | 2.16E-23 |
|  |  | HRV-C51 | HRV-C15 | 4768 | 4884 | RG**B**S | 2.01E-23 |
|  |  | HRV-C51 | HRV-C15 | 4946 | 5004 | RG**B** | 6.30E-30 |
|  |  | HRV-C51 | HRV-C15 | 5181 | 5245 | **R**GB | 4.73E-17 |
|  |  | HRV-C51 | HRV-C15 | 5423 | 5748 | RG**B**S | 1.82E-23 |
|  |  | HRV-C51 | HRV-C15 | 6074 | 6372 | RG**B**MCS | 7.11E-23 |
|  |  | HRV-C51 | HRV-C15 | 6850 | 6969 | RG**B**S | 3.66E-09 |
| 38 | HRV-C51[JF317015] | HRV-C2 | Unknown | 0 | 617 | **R**GBMS | 7.79E-19 |
| Subpopulation C2 | | | | | | | |
| 39 | HRV-C6[EF582387] | Unknown | HRV-A101-v1 | 11 | 599 | R**B**MCS | 2.76E-13 |
| 40 | HRV-C06[JN990702] | Unknown | HRV-A101-v1 | 11 | 599 | R**B**MCS | 2.76E-13 |
| 41 | HRV-C10[GQ323774] | Unknown | HRV-A101-v1 | 124 | 270 | R**B**MCS | 2.76E-13 |
| 42 | HRV-C3[EF186077] | Unknown | HRV-A101-v1 | 23 | 309 | R**B**MCS | 2.76E-13 |
| 43 | HRV-C1[EF077279] | Unknown | A65 | 35 | 168 | R**B**MCS | 1.76E-15 |
| 44 | HRV-C7[DQ875932] | Unknown | HRV-A101-v1 | 13 | 621 | R**B**MCS | 2.75E-13 |
| 45 | HRV-C43[JX074056] | Unknown | HRV-A101-v1 | 13 | 166 | R**B**MCS | 2.75E-13 |
| 46 | HRV-C(LZ651)[JF317016] | Unknown | HRV-A101-v1 | 11 | 498 | R**B**MCS | 2.76E-13 |

The table represents the data on 46 recombinant strains alongwith their potential major and minor parents obtained using RDP4 with p<0.00001. The recombinant strains alongwith their respective subpopulations (as obtained by the STRUCTURE program) are enlisted accordingly. The breakpoint positions are reported according to the position in the respective recombinant sequence. The recombination is detected using at least two of the six recombination detection methods in RDP4 program namely RDP, GENCONV, BOOTSCAN, MAXCHI, CHIMAERA, SISCAN and 3SEQ and are represented as R, G, B, M, C, S and Q , respectively. The method showing comparatively lowest p-value is shown and is represented as bold and the corresponding p-value is also reported.
